# A Low-Friction Capsule Robot with Drive–Control–Sensing Integration for Gastrointestinal Lesion Detection

**DOI:** 10.34133/research.0807

**Published:** 2025-08-04

**Authors:** Ziying Wang, Hang Yin, Zilin Wei, Shisheng Chen, Jiameng Li, Jingyao Sun, Wei Zhao, Yejuan Jia

**Affiliations:** ^1^ School of Disaster and Emergency Medicine, Tianjin University, Tianjin 300072, PR China.; ^2^ School of Mechanical Engineering, Hebei University of Technology, Tianjin 300401, PR China.; ^3^ Academy of Military Medical Sciences, Tianjin 300050, PR China.; ^4^ Tianjin Children’s Hospital, Tianjin University, Tianjin 300204, PR China.; ^5^ School of Acupuncture, Moxibustion and Tuina, Hebei University of Chinese Medicine, Shijiazhuang, Hebei 050200, PR China.

## Abstract

pH and temperature in the gastrointestinal (GI) tract are correlated with many diseases, but patients suffer from the pain of traditional testing methods. Functionalized capsule robots for GI tract sensing offer a potential approach for early GI disease diagnosis. However, most capsule robots struggle with control and sensing integration. In addition, the friction encountered by capsule robots during navigation poses a challenge for their application. Here, a capsule robot with a low coefficient of friction is proposed to continuously detect pH and temperature. The hydrogel skin reduces the surface friction coefficient by more than 4 times and achieves an average propulsion speed of 12.79 mm s^−1^ in a living porcine small intestine. The capsule system enables continuous detection over a pH range of 2 to 8 and a temperature of 36 to 40 °C. We demonstrate capsule robots’ capability of multimodal locomotion through complex stomach and small intestine environment. It solves the problem that capsule robots used for sensing functions cannot be precisely controlled and achieves drive–control–sensing integration.

## Introduction

The gastrointestinal (GI) tract is one of the largest hormone-secreting organs in the human body [1]. Poor dietary patterns and a range of triggers can lead to prevalent clinical conditions, including gastric and duodenal ulcers, functional dyspepsia, gastroesophageal reflux disease, and inflammatory bowel diseases like Crohn’s disease and ulcerative colitis [2–4]. Apart from endoscopy, monitoring of pH value and temperature is also an essential auxiliary diagnostic criterion. Therefore, the pH of affected GI regions is regularly tracked to assess the disease status in GI disorders and deliver treatment-oriented clinical evidence [5,6]. With the advancement of clinical medical technology, the development of flexible endoscopes and capsule robots for sensing have increased the ease of pH and temperature detection. However, both traditional flexible endoscopy and swallowed capsule robots for pH testing have considerable limitations and are in dire need of innovative breakthroughs: The traditional clinical monitoring method is insertion of a pH meter probe into the stomach through endoscopy for pH detection [7,8]. This method can cause intense discomfort to the patients [9]. Although capsule robot detection for pH sensing allows for wireless, noninvasive, and painless diagnostic procedures, its movement is usually dependent on bowel peristalsis, which makes it susceptible to leakage and misdetection. The domain of medical micro-robotics is progressing rapidly, primarily aimed at tackling a variety of biomedical challenges within the human body [10–13]. The convergence of traditional capsules with advanced micro-robotic systems enables transformative advances in innovative diagnostic and therapeutic approaches.

Existing research classifies actuation mechanisms into passive and active categories. Research on passive capsules has focused on the design of microelectromechanical systems, which add sensing capabilities, but their motion is not controllable [14]. Compared to passive capsule robots, active capsules can provide richer biomedical functions, such as targeted drug delivery and biopsy taking [15–18]. Primary actuation methods employed in contemporary active capsule robots encompass pneumatic and hydraulic systems [19], electromechanical [20,21], shape memory alloys [22], and magnetic actuation [23–25]. Among these methods, magnetic actuation exhibits superior technological adaptability [26]. Additionally, it provides notable benefits, including non-contact operation, high controllability, and excellent penetration performance [27,28]. Consequently, magnetic actuation is regarded as one of the safest and most effective methods for capsule applications. The development of magnetic capsule robots has instilled considerable confidence in biomedical applications [29].

At present, current magnetic capsule robots have limited application scenarios, lacking additional sensing functionalities. The fact that magnetic drive technology inherently offers the synergistic benefits of control and perception provides a natural platform for multifunctional integration. Many capsule robots for pH detection have now been reported [30–32]. However, all of them are passive and cannot be driven or have their kinematic states controlled by personnel, due to safety size limitations, which cannot meet the integrated design requirements of control and sensing. Therefore, the research on magnetic field actuated control sensing integrated capsule robot to achieve multi-parameter dynamic monitoring of the target area through precise navigation by magnetic field not only overcomes the randomness defects of passive detection, but also provides an innovative solution for precision medical treatment of GI diseases. In addition, the friction encountered during intestinal movement has been a major challenge for capsule robots, and the frictional resistance of capsule robot is related to the surface coefficient of friction (CoF) [33]. Some studies have sought to enhance the locomotion speed of capsule robots by optimizing their mechanical structures or leveraging surface resistance to increase frictional interactions [34–36]. However, the thickness of the intestinal wall makes it highly susceptible to secondary damage during inspection [37–39]. Therefore, the development of low-friction capsule robots with integrated control and sensing is of great research significance.

In this study, we propose a magnetically actuated capsule robot with low CoF for continuous detection of pH and temperature. The modular design increases the controllability of the detection by encapsulating the pH and temperature sensors in the detection module and driving the movement of the capsule by generating a responsive magnetic field from the capsule’s drive module. The capsule surface is modified by covalent cross-linking hydrogel skin, which reduces the friction coefficient and improves the motion efficiency of the capsule robot. Meanwhile, we proposed a multimodal motion to adapt to the complex environment of the GI tract. The capsule system was subjected to in vitro sensing simulations using artificial digestive fluids, and the integration of the drive sensing of the capsule was verified in in vitro biological experiments. The comprehensive performance evaluation of the capsule system lays the foundation for future clinical evaluations.

## Results

### Design of a capsule robot

**Fig. 1.**
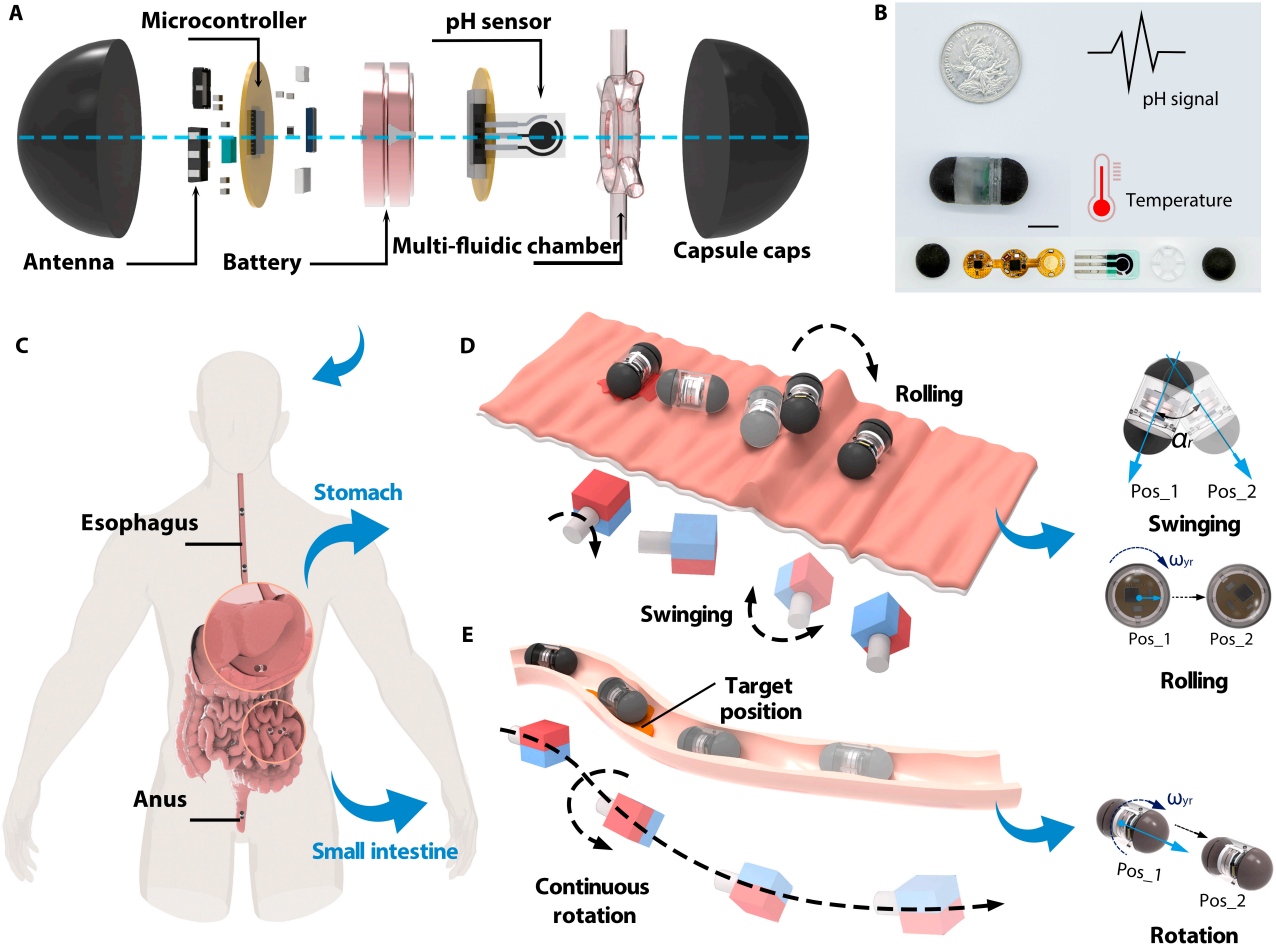
Design and motion modes of the capsule robot. (A) The capsule robot in exploded 3-dimensional view. From right to left, the capsule consists of a drive module that generates a responsive magnetic field, a multi-fluidic chamber, a pH sensor, a button cell, and a flexible electrochemical analysis circuit that incorporates a temperature sensor. GI fluid is collected by the multi-fluidic chamber, which generates a potential difference in contact with the working electrodes, and the detected potential signals and temperature are processed by the built-in detection circuit and transmitted wirelessly to a mobile device. (B) Photograph of the size and details of the capsule robot. Scale bars: 10 mm. (C) Schematic of the human GI tract and capsule robot. (D) Schematic of the multi-multimodal locomotion in the stomach. (E) Schematic of the propulsion modal in the intestine.

We chose hemispherical capsule heads and smooth surface for the robot design because such a shape can optimize the flow field distribution and reduce the local contact stress with the GI tract [40–42]. Furthermore, we divide the capsule robot into a drive module and a sensing module through a modular design (Fig. 1A). This design ensures integration of sensing and actuation capabilities in a limited size. The fabrication details of the capsule robot are described in Note S1.

The sensing module system comprises a multi-fluidic chamber, a pH sensor, a button cell, and a flexible electrochemical analysis circuit that incorporates a temperature sensor. After the GI fluid is collected by the multi-fluidic chamber, the system works in tandem to detect pH and temperature. The capsule diameter is 7 mm, approximately equivalent to a standard size 2 capsule (Fig. 1B). The capsule robot achieves coordinated movement through the integrated actuation of its driving and sensing module. The system employs a 7 degrees of freedom (7-DOF) robotic arm with a rotating NdFeB permanent magnet (50 mm length) to magnetically manipulate the device in 3-dimnensional (3D) space (Fig. S1), with dual-magnetic capsule heads as the internal driver and a cubic permanent magnet as the external driver.

The robot is swallowed and reaches the stomach and intestines, and is finally expelled from the body through the anus (Fig. 1C). However, the capsule robot navigating in the GI tract will face different surface environments; for example, the rugal folds of the stomach wall create physical barriers that impede robotic movement, and the narrow environment of the intestinal tract creates a degree of freedom restriction and frictional resistance. Thus, the capsule robot can achieve swinging, rolling, and continuous rotational motions under the control of permanent magnets to increase GI tract environmental adaptability. For instance, on rugal gastric folds, the robot can combine swinging and rolling motion to overcome raised surface (Fig. 1D). In the narrow small intestine, the robot is propelled by continuous rotation to convert fluid resistance into radial force on the capsule body (Fig. 1E) [14,36,43].

### Detection module system design

**Fig. 2.**
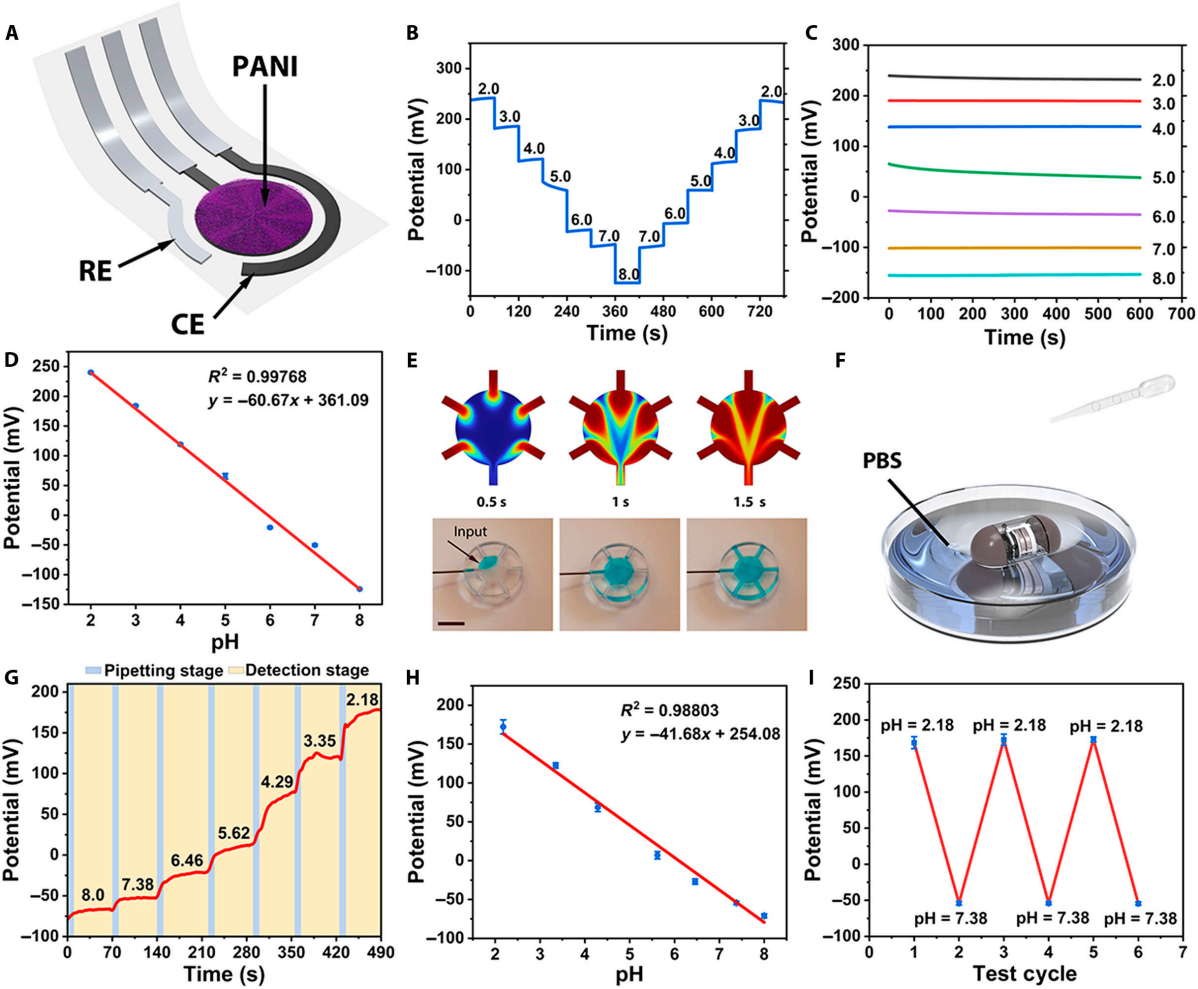
Fabrication and electrochemical measurements of the pH detecting system. (A) Schematic of the pH sensor. (B) Potentiometric response of the pH sensor in different PBS buffer solution. (C) The sensitivity of the pH sensor with pH ranging from 2.0 to 8.0. (D) The initial linear fitting curve of the pH sensor. (E) Simulations and photographs of solution dispersion in a multi-fluidic chamber at different times (inlet flow rate, 1 ml min^−1^). Scale bars, 5 mm. (F) Schematic diagram of capsule robot system testing. (G) Potentiometric response of the pH sensors in different PBS buffer solution, real-time calibration by pH meter. (H) Redefined the linear fitting curve of the pH sensor. (I) Five alternating detections in pH value 2.18 and 7.38, and maximum relative standard deviations of 8.31 and 3.56 mV in its potential response, respectively.

The pH electrode was prepared by screen printing, which was flexible and bendable. The working electrode’s surface area was ~12.6 mm^2^; therefore, it could be easily connected to the multi-fluidic chamber. Polyaniline was used to modify the working electrode (Fig. 2A). Systematic characterization experiments were conducted to evaluate the performance of the pH sensor. Based on the physiological pH range of the intestinal fluid reported in previous studies [44], phosphate-buffered saline (PBS) buffer solutions spanning pH 2 to 8 were utilized to assess the sensor’s response across its pH-sensitive detection range. Figure 2B shows the open-circuit potential-time (OCP-t) responses of the pH sensor in a buffer with a gradient from 2 to 8, which can respond promptly to intestinal fluid pH changes. During the continuous 60-s detection time in each PBS buffer solution, the potential response of the pH sensor remained stable, whose maximum potential drift was 17.22 mV. Figure 2C shows the OCP-t curves of the pH sensor in PBS solutions within the pH range of 2 to 8. The results demonstrate that within a testing period of 600 s, the sensor exhibits a robust and stable response to buffers of varying pH values. Before each detection, the pH meter was used to calibrate the true pH of the buffer. Therefore, the baseline response potential can be defined. In this way, a linear fitting curve could be defined (*N* = 6) (Fig. 2D). According to the linear fitting curve, the response sensitivity of the pH sensor could reach ~−60.67 mV pH^−1^ with a correlation coefficient (*R*^2^) of ~0.99768.

The multi-fluidic chamber serves as an intermediary between the electrodes and GI fluids, preventing direct contact between the fluids and the capsule robot. The time it takes for the solute concentration in the reservoir to adjust to the new concentration is a key performance indicator [45]. The refresh time of the module was determined via numerical simulations. The simulation and experimental results indicate that the refresh time is approximately 2 s (Fig. 2E).

The assembled capsule robot was placed in PBS buffer solution to test the stability of the system for pH detection (Fig. 2F). The capsule robot was placed in an initial pH 8.0 PBS buffer solution followed by a drop of pH 2.0 PBS buffer and waited for the capsule to respond 10 s after the drop. Figure 2G shows the image resulting from the denoising process, demonstrating that the response potential shows an increasing trend during the drop-addition phase, and the response potential is gradually stable during the detection phase that lasts for 60 s. Therefore, the baseline response potential was redefined. In this way, a new linear fitting curve could be defined (*N* = 5) (Fig. 2H). The response sensitivity of the pH sensor in the capsule robot could still reach ~−41.68 mV pH^−1^ and *R*^2^ could still reach ~0.99768. Figure 2I shows the OCP-t response of the pH sensor alternately at pH 2.18 and pH 7.38 in PBS buffer. Based on the findings from the detection conducted in an extreme pH environment across 5 alternating cycles, the pH sensor exhibited maximum relative standard deviations of 8.31 and 3.56 mV in its potential response, respectively. These researches demonstrate the capsule system’s ability to perform effectively in pH detection within the GI tract.

**Fig. 3.**
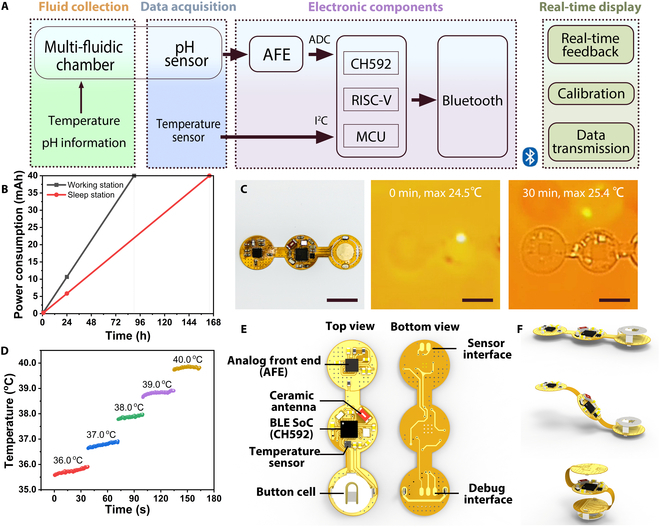
Circuit design and characteristics. (A) Description diagram of the detection circuit system. (B) Power supply from a 40-mAh battery in 2 modes. (C) Thermal images of the circuit board after various times of operation. Scale bars: 10 mm. (D) Temperature change measured by the capsule robot from 36 to 40 °C. (E) Illustration of internal PCB circuits. (F) Schematic of flexible PCB folding.

Most in vivo pH detection devices require external wiring and are limited to laboratory use, while a low-power wireless alternative could make in vivo detection more convenient [46]. The sensor data acquisition section of our ingestible capsule is primarily composed of 4 main components: a multi-fluidic chamber for fluid collection, a pH sensor prepared by screen printing, a flexible circuit designed for wireless data acquisition and transmission, and an upper computer for information reception. A RISC-V-based microcontroller (MCU) equipped with a Bluetooth Low Energy wireless communication protocol serves as the primary control unit. The temperature sensor transmits data to the MCU via the I^2^C protocol, and the pH data are sampled using a 12-bit on-chip analog-to-digital converter (ADC). The acquired signals are then processed and visually displayed by the upper computer for further analysis (Fig. 3A).

To extend the operational lifespan of the detection capsules, the system was engineered to feature distinct sleep and work modes, with the circuits predominantly operating in the sleep mode. The button cell of 40 mAh can provide power for 164 h in a sleep station and 90 h in a working station theoretically in each state (Fig. 3B). The temperature field distribution of the circuit was designed to minimize the temperature rise to less than 1 °C, thereby reducing the impact of circuit-generated heat on biological tissue (Fig. 3C).

 The temperature sensor was tested within the human body temperature range after calibration, and the sensor exhibited a response deviation within ±0.5 °C (Fig. 3D). The electronic components within the capsule were integrated onto a single flexible circuit board. Using commercially available flexible printed circuits (FPCs) with a custom-designed island–bridge structure, the diameter of the islands is 12 mm. It integrates an MCU chip with a built-in DC–DC converter, which can manage power more efficiently by dynamically adjusting the power supply according to the MCU’s requirements, thereby improving energy efficiency. The board also includes a ceramic antenna for wireless signals, a pH analog front-end for sensor signal acquisition, and a selection of capacitors and resistors for circuit stability. A button cell battery is used for power supply (Fig. 3E). The island–bridge structural design endows the circuit with a foldable feature, allowing electronic components to fit inside a capsule (Fig. 3F).

### Low friction of hydrogel skin

**Fig. 4.**
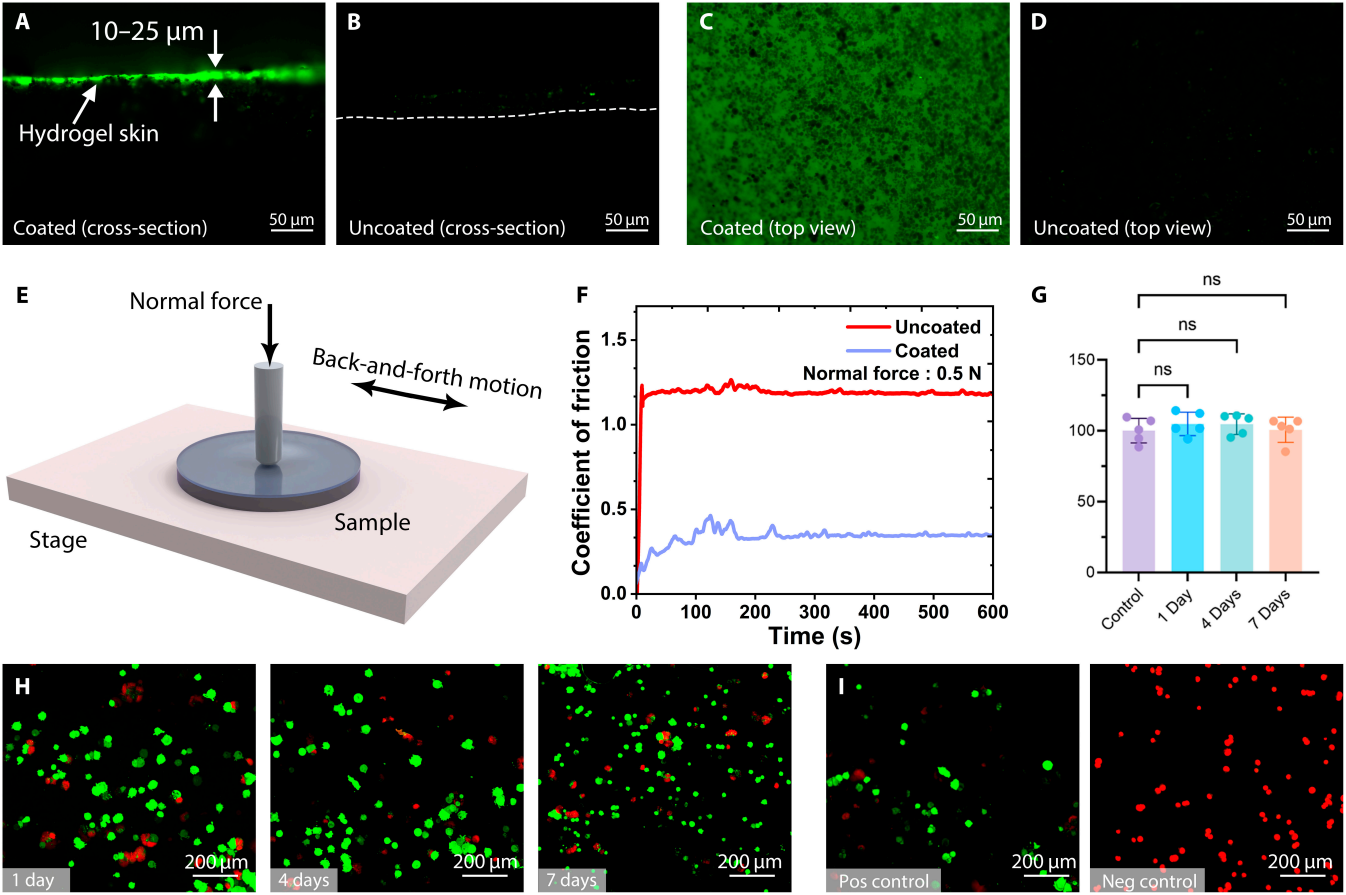
Characterization and biocompatibility of hydrogel skin. (A and B) Cross-sectional view of the coated and uncoated samples of Ecoflex 00-30/NdFeB (50 wt%); the hydrogel coating thickness is 10–25 μm. The dashed line in (B) indicates the boundary of the cross-section of the uncoated sample. (C and D) Top view of the coated and uncoated samples. The fluorescing specks visible in the uncoated sample were due to residual fluorescein adsorbed onto the surface. All scale bars: 50 μm. (E) Schematic of the experimental setup designed for measuring friction coefficients with a friction-abrasion testing machine. (F) Semi-logarithmic plots of the CoF of coated and uncoated specimens measured over time during friction testing of the specimens with and without coating under a normal force of 0.5 N at a reciprocating motion frequency of 1 Hz. (G) Assessment of Caco-2 cell viability via a hydrogel-conditioned medium with differing conditioning durations, as evaluated by CCK-8. The values represent the mean ± SD (*n* = 5). (H) Evaluation of the biocompatibility of the samples through a live/dead assay on Caco-2 cells, cultured in a conditioned medium with conditioning periods of 1, 4, and 7 days. After culture, the Caco-2 cells were stained with calcein-AM (green) and ethidium homodimer-1 (red) to visualize the viable and dead cells, respectively. Scale bars: 200 μm. (I) Positive/negative control live-dead cell staining of Caco-2 cells. Scale bars: 200 μm.

The hydrogel skin was synthesized through cross-linked hydrophilic polymers (polyacrylamide [PAM]). We used the same hydrogel solution preparation method based on our previous work [47]. Microscopic characterization conclusively demonstrated the successful formation of hydrogel skin on treated samples (Fig. 4A to D). Comparative fluorescence microscopy analysis of planar specimens (1-mm-thick sheets) quantitatively determined the hydrogel coating thickness as 10 to 25 μm, with measurements derived from contrast imaging between coated and uncoated surfaces.

The polymerized hydrogel skin significantly reduced the surface friction of the robot. The mechanical stability and CoF of the hydrogel skin were evaluated using the fabricated specimens (Fig. 4E). The measurements revealed a 4-fold decrease in CoF under a load of 0.5 N compared to the uncoated sample, demonstrating the lubricating effect of the hydrogel skin under all tested conditions. Additionally, after 600 s of continuous measurement, the CoF stabilized, with the coated sample maintaining a lower stable CoF than the uncoated sample. This indicated that the hydrogel skin not only effectively reduced the friction coefficient but also exhibited considerable mechanical stability (Fig. 4F).

We then analyzed the cytotoxicity of PAM hydrogel skin using the Caco-2 cell line, which has been widely used as a model of the epithelial barrier on the intestine [46]. Leaching experiments were conducted by incubating samples in fresh culture media for 1 to 7 days to collect any released chemicals. Caco-2 cells were subsequently exposed to the conditioned media containing the released compounds for 2 days, and cell viability was assessed using live/dead cell staining. As shown in Fig. 4G and H, no significant difference in cell viability was observed between cells maintained in pristine culture medium (positive control) and those exposed to media conditioned with the PAM hydrogel skin for 1 to 7 days. The viability of Caco-2 cells in the experimental group remained comparable to that of the control group, demonstrating the excellent biocompatibility of the capsule robot (Fig. 4I). For detailed evaluation and improvement of biocompatibility, please refer to Note S2.

### Control strategy analysis

**Fig. 5.**
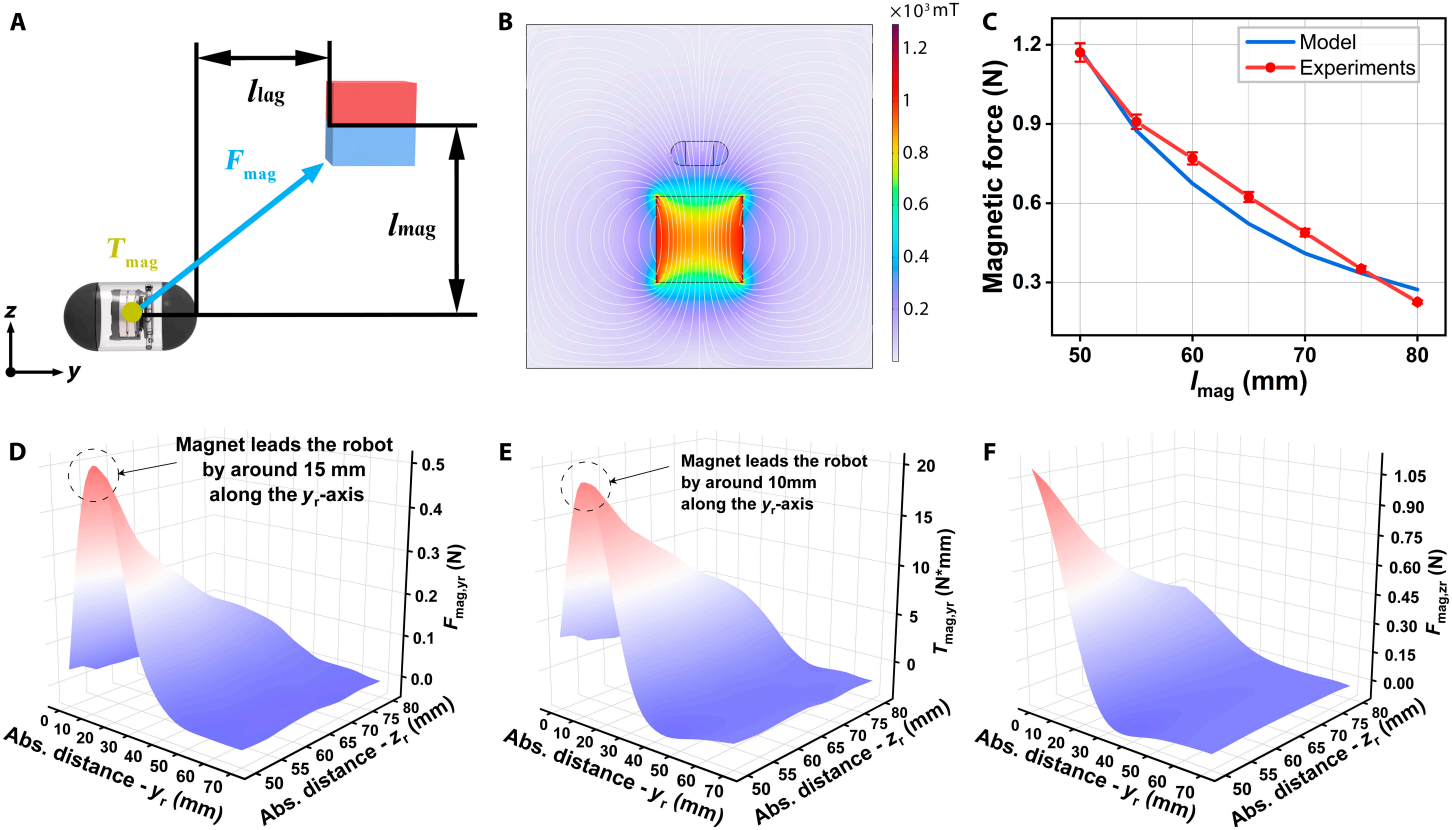
Relationship of magnetic coupling. (A) Illustration of essential variables. (B) Simulation and analysis of static magnetism of magnets and capsule robots. (C) Quantification of the magnetic force received by the capsule robot along the *z*_r_-axis. The data are presented as mean values ± standard deviation for *n* = 5. (D) Variation of the maximum magnetic force along the *y*_r_-axis *F*_mag,*y*r_ for different distances between the robot and the magnet along with the *y*_r_ -and *z*_r_-axis. Maximum *F*_mag,*y*r_ is achieved when the magnet leads the robot by around half of the size of the robot, i.e., 15 mm. (E) Variation of magnetic torque around the *y*_r_-axis *T*_mag,*y*r_. Maximum *T*_mag,*y*r_ is achieved when the magnet is 10 mm ahead of the robot. (F) Variation of magnetic force along the *z*_r_-axis *F*_mag,*y*r_.

The robotic actuation system employs both magnetic torques *T*_mag_ and forces *F*_mag_ for precise control. There are 2 essential variables in the motion of the capsule robot: (a) distance between the magnet and the robot along the *z*_r_-axis *l*_mag_ and (b) distance of robot’s lag to magnet *l*_lag_ along the *y*_r_-axis (Fig. 5A). Assume that the capsule robot is moving straight along the *y*-axis and *l*_lag_ is fixed, the variable *l*_mag_ in the *z*-axis direction will directly affect the *F*_mag_ and *T*_mag_ along the *y*-axis. When the capsule robot is steering toward the *x*_r_-axis, at this time *l*_mag_ is fixed, and the variable *l*_lag_ in the *y*-axis direction directly affects the magnitude of the *F*_mag_ and *T*_mag_ of the capsule robot along the *y*-axis.

To explain the observed behaviors, a simulation model was developed in COMSOL Multiphysics 6.1 to quantify the *F*_mag_ subjected by the capsule robot at *l*_lag_ = 0 for different *l*_mag_ cases, and the magnet produces a maximum flux density of 180 mT (Fig. 5B and Fig. S3). As illustrated in Fig. 5C, *F*_mag_ to which the capsule robot is subjected decreases linearly as *l*_mag_ gradually increases. This is due to the fact that the attenuation of the magnetic field decreases exponentially by increasing distance [48].

When the parameter *l*_mag_ is fixed, we quantify the *F*_mag_ and *T*_mag_ that the robot is subjected to for *l*_lag_ from 0 to 70 mm (Fig. S4). As illustrated in Fig. 5D, when the capsule robot moves along the *y*-axis in a spatial magnetic field, the *F*_mag,*y*r_ to which the capsule robot is subjected by the magnet peaks at 50 mm along the *z*_r_-axis of the capsule robot’s spatial coordinates and 15 mm along the *y*_r_-axis. Figure 5E shows that the magnet is subjected to *T*_mag,*y*r_ peaks at 50 mm along the *z*_r_-axis and 15 mm along the *y*_r_-axis of the capsule robot’s spatial coordinates. Figure 5F illustrates that the *F*_mag*,z*r_ subjected to the capsule robot in the *z*-axis decreases with the increase of *l*_mag_ and *l*_lag_. In summary, the control distance at which the magnet can produce the best response from the capsule robot should be at *l*_mag_ = 50 mm and *l*_lag_ = 15 mm. Therefore, when the capsule robot is in a turning position or in a position with a large reaction torque *T*_react_, the distance between the capsule robot and the magnet should be set to be between 10 and 15 mm, and the *l*_mag_ should be reduced in order to allow the capsule robot to obtain a larger *T*_mag_ to overcome the *T*_react_. For detailed quantification of the reaction force *F*_react_ and reaction torque *T*_react_, please refer to Note S3.

**Fig. 6.**
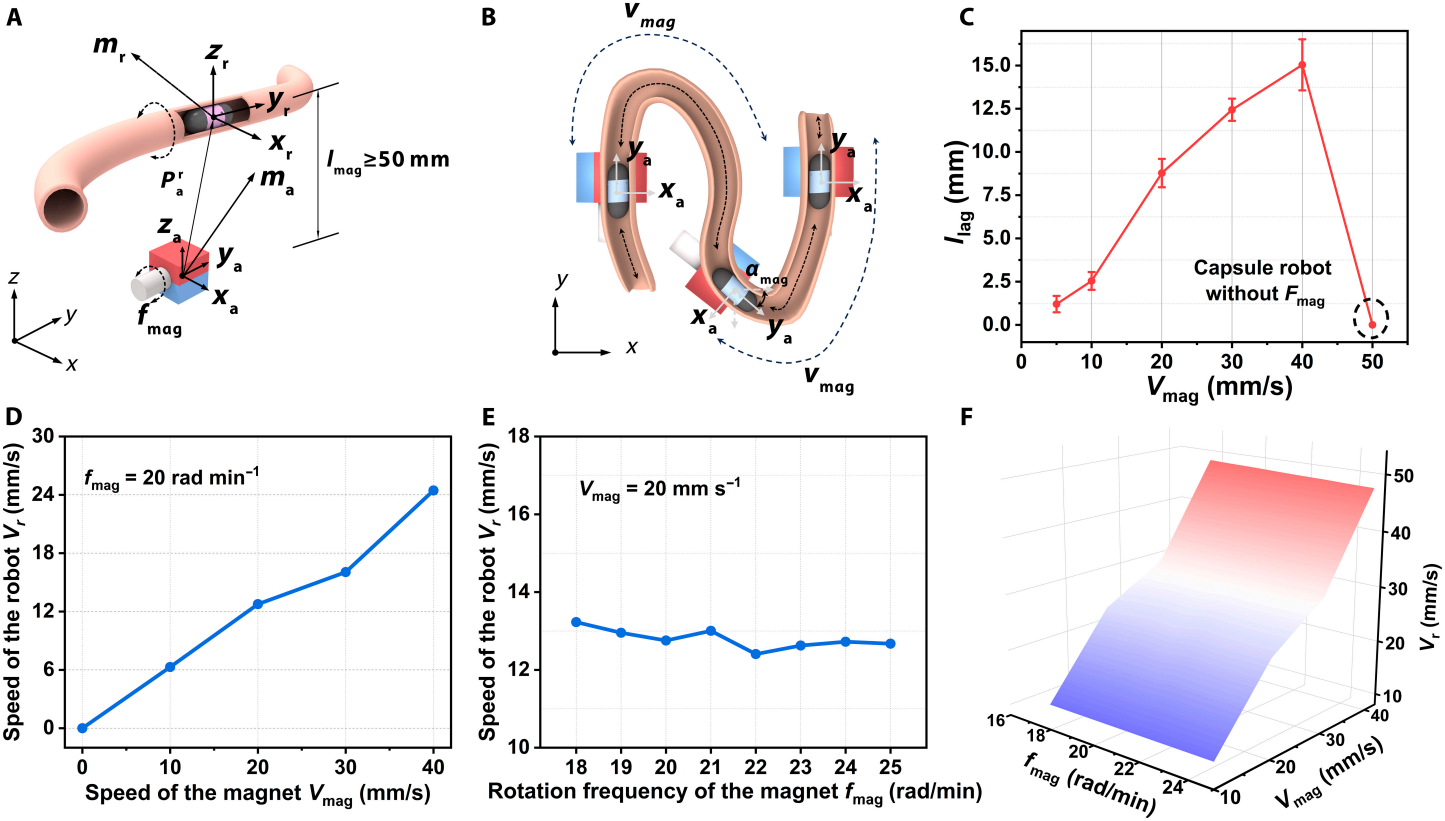
Relationship of *v*_mag_−*v*_r_. (A) and (B) are schematic diagrams of the actuation strategy of the robot. (C) Effect of the *v*_mag_ to *l*_lag_; capsule robot will stop moving when *v*_mag_ > 40 mm s^−1^. (D) Robot speed *v*_r_ along with the traversing speed of the magnet *v*_mag_. The rotation frequency *f*_mag_ was set to be 20 rad·min^−1^. (E) Robot speed along with *f*_mag_ and *v*_mag_ was set to be 20 mm s^−1^. (F) Relation between robot speed *v*_r_ and *f*_mag_, *v*_mag_.

The movement speed of the capsule robot is determined by the magnet. Therefore, the capsule robot is propelled by 4 basic variables: (a) traversing speed of the magnet *v*_mag_ along the *y*-axis, (b) rotation frequency of the magnet *f*_mag_, (c) traversing speed of the robot *v*_r_ along the *y*_r_-axis, and (d) distance of robot’s lag to magnet *l*_lag_ along the *y*_r_-axis. Specifically, the magnet is rotated around the *y*_a_-axis at a frequency *f*_mag_ (Fig. 6A), translated ahead of the robot at a speed of *v*_mag_, and reoriented by an angle of *α*_mag_ to align with the *y*_a_–*z*_a_ plane relative to the current trajectory (Fig. 6B). In the *v*_mag_–*v*_r_ relationship, the effects of varying traversing speeds *v*_mag_ and frequencies *f*_mag_ are quantified using a 200-mm straight porcine small intestine model, with *v*_mag_ ranging from 10 to 40 mm s^−1^ (Movie S1).

We first discussed the relationship between *v*_mag_ and *l*_lag_. As shown in Fig. 6C, the capsule robot is not driven by the magnet when the propulsion speed of the magnet is greater than 40 mm s^−1^, i.e., *F*_mag,*y*r_ and *T*_mag,*y*r_ = 0 along the *y*_r_-axis, and the experimental results are consistent with the simulation results (Fig. S4B). When *v*_mag_ ≤ 10 mm s^−1^, the perpendicular distance *l*_lag_ between the magnet and the capsule robot in the *y*–*z* plane is minimized, i.e., the robot advances almost immediately after the magnet, and the mean standard deviation of *l*_lag_ is ±0.48 mm for *n* = 6. When the advancement speed of the magnet was 40 mm s^−1^, the perpendicular distance between the magnet and the capsule robot in the *y*–*z* plane increased to 15 mm. Within 6 replicate experiments, the average standard deviation of *l*_lag_ was ±1.47 mm. The results illustrate that when *v*_mag_ ≤ 10 mm s^−1^, although the robot can be ensured to advance at a stable speed, the motion efficiency is low, which affects the overall inspection time. When *v*_mag_ ≥ 40 mm s^−1^, *l*_lag_ increases and the capsule robot is out of the control of the magnet, which is unfavorable for the motion control of the capsule robot. Therefore, the effective manipulation speed interval of the magnet is [10,40] mm s^−1^.

Secondly, the relationship between *v*_mag_, *v*_r_, and *f*_mag_ is studied. When *f*_mag_ is fixed to 20 rad min^−1^, the advancing speeds of setting *v*_mag_ are 10 to 40 mm s^−1^. When *f*_mag_ is fixed, the larger the magnet advancing speed *v*_mag_ is, the faster the capsule robot advances, up to a maximum of 24 mm s^−1^ (Fig. 6D). When *v*_mag_ = 20 mm s^−1^, *f*_mag_ is set from 18 to 25 rad min^−1^. The capsule robot does not differ much in advancing speed, with an average of 12.83 mm s^−1^ (Fig. 6E). The data demonstrate that *f*_mag_ contributes less significantly to the robot’s velocity *v*_r_ compared to *v*_mag_. The robot achieves an average velocity *v*_r_ of approximately 12.79 mm s^−1^ at *v*_mag_ = 20 mm s^−1^. It should be noted that *v*_r_ exhibits variability due to fluctuations in magnetic forces and torques, and the reported values represent path-averaged measurements.

With both *v*_mag_ and *f*_mag_ as variables, the 3D plotting illustrates the *f*_mag_–*v*_mag_–*v*_r_ matching relationship more directly (Fig. 6F). When *v*_mag_ is lower and *f*_mag_ is higher, *v*_r_ is smaller, at which time the distance of *l*_mag_ is controlled to obtain a larger *T*_mag_, and such a strategy can be used to enable the capsule robot to pass through the intestines with a smaller radius of curvature; when *v*_mag_ is higher and *f*_mag_ is smaller, *v*_r_ is higher, and such a actuation strategy can be used for the capsule robot to maintain a higher speed of movement to advance through the intestines. Notably, Fig. 6F also illustrates that the direct effect of *f*_mag_ on *v*_r_ is small, which is consistent with the conclusions drawn from controlling the *v*_mag_ and *f*_mag_ variables to study *v*_r_.

**Fig. 7.**
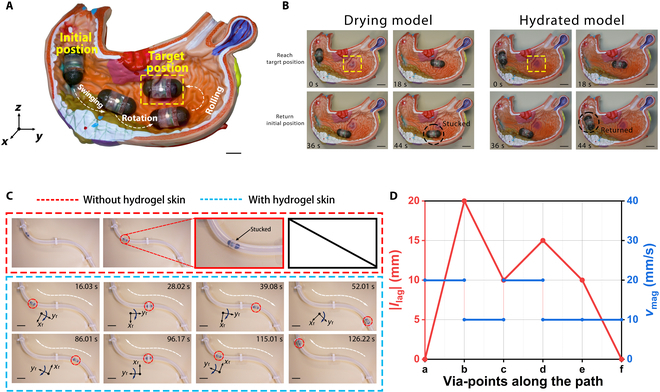
Motion analysis of the capsule robot. (A) Three multimodal motion coordination actuations for capsule robots. From the initial position to the target position, it is swinging, continuous rotation, and rolling, respectively. Scale bar: 10 mm. (B) Motion testing of capsules in the drying gastric model and hydrated model, respectively. Scale bars: 10 mm. (C) Motion testing of capsules in the tube model. Scale bars: 40 mm. (D) Strategies for tube model traversing.

The capsule robot can complete the multimodal motion modes of swinging, continuous rotation, and rolling under the control of magnet. The combination of different motion modes can be realized to move in different environments of the stomach and intestines. In the stomach model, the capsule robot can reach the target position from the initial position by a combination of 3 motions (Fig. 7A and Movie S2). Multimodal motion of capsule robot is illustrated in Note S4.

To investigate the efficiency of robot locomotion under the influence of hydrogel skin, we tested it through 2 different environments with the same motion illustrated in Fig. 7A. We set *l*_mag_ = 50 mm and *v*_mag_ = 15 mm s^−1^, and the capsule robot took 24 s to complete the 3 phases of motion in the drying model, but was stuck during the return stage, which was due to the resistance *F*_react_ > *F*_mag_ generated by the drying model, and *T*_react_ caused the capsule robot to be stuck. When the capsule robot was in the hydrated model, it successfully completed the expected movement in 24 s (Fig. 7B). This was caused by the hydrogel skin increasing the hydrophilicity and decreasing the CoF, which made *F*_react_ < *F*_mag_. Experimental data confirm robust motion controllability and environmental adaptability of the capsule robot, with consistent performance maintained under challenging conditions such as low-friction interfaces and variable mucosal resistance (Movie S3).

Continuous rotational motion in lumen testing further illustrates the effect of hydrogel skin on the efficiency of capsule robot motion. The robot is maneuvered through a hydrated narrow tube (Ø16 mm) in bidirectional motion, with its trajectory discretized into via-points (a) to (d) along a curvature radius of *R*_c_ = 150 mm (Fig. S5). The uncoated robot experiences mechanical arrest in (b) to (c) due to insufficient hydrophilicity. Despite omnidirectional steering capability enabling precise distal tip orientation, the substantial resistance prevents forward progression. Subsequent testing with the coated robot demonstrates markedly improved performance, as the hydrophilic coating reduces interfacial friction, enabling effective locomotion (Fig. 7C). Notably, the observed translational velocity is lower than theoretical predictions (9.61 mm s^−1^), due to dimensional variations in the manually fabricated S-shaped tube, where localized diameter reduction increases frictional resistance. These results demonstrate the capsule robot’s potential for diagnostic applications in narrow, tortuous luminal environments. Complete navigation strategies are detailed in Fig. 7D.

**Fig. 8.**
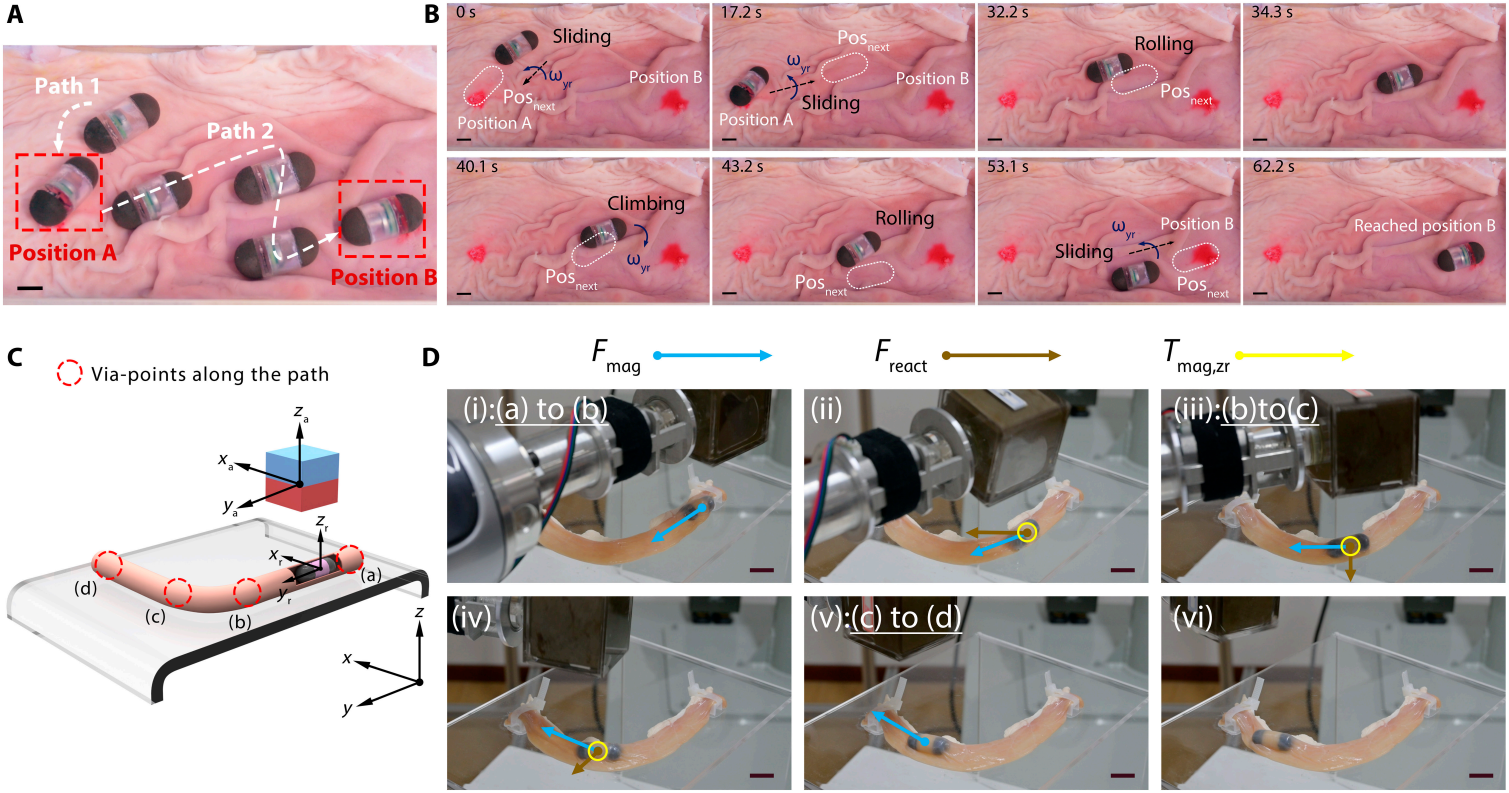
Demonstration of multimodal locomotion in ex vivo porcine stomach and small intestine, respectively. (A) Demonstration of multimodal motion of capsule robot in a folded gastric wall. (B) Illustration of multimodal movement strategies of capsules in the folded gastric wall. Scale bars: 10 mm. (C) Schematic of discretizing via-points traversed by curvilinear routes. (D) Snapshots for curved routes traversing with *R*_c_ = 50 mm. The magnet generates a magnetic suction force and a magnetic moment that brings the robot into contact with the intestinal wall on one side, and the reaction force *F*_react_ from the luminal wall causes the robot to slide along the intestinal wall, enabling the traversal of a curved route. Scale bars: 10 mm.

In ex vivo porcine stomach experiments, 2 target lesion sites are established to evaluate the capsule robot’s diagnostic capabilities. The robotic system demonstrates successful navigation from target position A to B, employing an identical actuation strategy to that implemented in the stomach model (Fig. 8A and Movie S5). The operational sequence consists of 4 distinct phases: (a) initial positive rotation to reach target position A, (b) swinging to enter the crease, (c) magnetic-guided forward propulsion along gastric folds to the inflection point, and (d) reverse rotation-mediated fold traversal followed by rolling motion to target site B (Fig. 8B). For detailed mechanistic analysis of capsule robots overturning gastric wall folds, please refer to Note S5.

The capsule robot’s intestinal navigation capability is validated using an ex vivo small intestine model, employing control strategies analogous to those developed for curved silicone tube environments. To analyze locomotion dynamics, the trajectory is discretized into sequential via-points (a) to (d) (Fig. 8C and Movie S6). During navigation through (b) and (c), magnetic field orientation is optimized such that the $y_{a}$-axis aligns parallel to the (c)-(d) path, simultaneously generating forward propulsion and positive $y_{r}$-axis rotation. This configuration produces 2 synergistic effects: 1) magnetic torque *T*_mag*,zr*_ aligns the robot’s *x*_r_-axis with the global *x*_a_-axis, and 2) magnetic force *F*_mag_ directs motion toward the (c)-(d) segment. The miniaturized capsule design enables efficient propulsion by overcoming fluidic resistance and intestinal wall friction. Upon intestinal wall contact, the resultant reaction force *F*_react_ provides additional directional guidance toward the (c)-(d) path, facilitating wall-adherent sliding motion. These biomechanical interactions are schematically illustrated in Fig. 8D with complete navigation strategies detailed in Fig. S6. For detailed quantification of the contributing torques, please refer to Note S6.

## Discussion

To address the limitations of sensing capsule robots lacking active controllability and the low locomotion efficiency of actively movable capsule robots, we have developed a low-friction magnetically controlled capsule robot and investigated its actuation strategy. The advantages of this device are demonstrated in the following aspects. First, in terms of accessibility, its controlled retrievable motion enhances maneuverability within the GI tract, eliminating the uncertainty in examinations caused by passive movement following peristalsis. Second, it ensures a lower CoF (maximum on the order of 0.3) compared to commercially available or reported capsule robots, resulting in a safe interaction between the capsule and the GI environment. Finally, multimodal motion increases its adaptability in the GI tract environment; e.g., a combination of rolling and swinging allows it to go over folds in the stomach wall, and continuous rotational motion increases its speed of movement in the small intestine (average of 12.79 mm s^−1^). The current robot design was compared with the relevant works in the literature, as shown in Table S2.

Beyond the medical functions demonstrated in this work, the modular design enables the development of additional potential medical applications. For instance, modifying the composition of the capsule robot’s driving module (e.g., Fe₃O₄ nanoparticles) could facilitate on-demand thermal hemostasis. Similarly, adapting the sensing module by integrating electrodes would allow for multimodal data acquisition or targeted drug delivery. Notably, given the robot’s ability to generate larger magnetic moments with decreasing distance and its low surface friction coefficient, its utility could potentially be extended to vascular environments, functioning as a controllable blood sampling device.

The current study has several limitations. First, endoscopy remains the most reliable tool for early screening of GI diseases, while pH monitoring primarily serves as an auxiliary diagnostic and localization tool [5,49,50], and integrating existing capsule endoscopy imaging modules into our sensing system could substantially enhance real-time monitoring capabilities during capsule procedures. Second, although we employed simplified force modeling and simulation analysis to explain the observed phenomena, the actual GI environment is considerably more complex. Future studies should account for additional factors such as intestinal geometry variations and mucosal resistance variability to ensure reliable in vivo operation of the robotic system. Finally, while our navigation experiments were conducted with visual feedback, practical clinical applications would require auxiliary positioning technologies such as CT, x-ray, ultrasound, or magnetic localization. Notably, magnetic positioning technology demonstrates particularly high compatibility with magnetically controlled capsule robots. Therefore, developing multi-magnetic-field localization technology represents a promising direction to advance clinical applications of capsule robotics, which will be a focus of our future research.

## Materials and Methods

### Fabrication of the pH sensor

Polyethylene terephthalate served as the foundational substrate for the pH sensor. Initially, Ag/AgCl conductive ink was deposited onto the surface of the exposed electrode, functioning as both the reference electrode and conductive pathways. Following a drying period at ambient temperature (~25 °C) for 30 min, carbon conductive ink was subsequently applied to the electrode, with careful attention to minimize overlap with the previously printed Ag/AgCl layer, thereby preserving the integrity of the electrical connections. The above procedure was repeated to ensure thorough drying of the carbon ink. Consequently, the modified bare electrode was prepared for further pH sensor fabrication. Then, cyclic voltammetry was performed for 25 cycles in a potential range of −0.2 to 1.2 V after immersing the working electrode in a 0.1 M aniline solution containing 1 M HCl (Fig. S2).

### Fabrication and characterization of the multi-fluidic chamber

The 3D-printed multi-fluidic chamber comprises a cylindrical reservoir and multiple inlet channels. The radius of the chamber is 4.6 mm with a thickness of 1.6 mm, while the inlet channels are 2.4 mm in length and 0.8 mm in width. The chamber refresh time was simulated and a 3D model was constructed based on the device geometry. Numerical simulations were conducted using COMSOL Multiphysics 6.1 finite-element software to solve the Stokes equation for incompressible flow. The solute concentration within the chamber was monitored by calculating the average concentration over the bottom surface of the chamber. The channel configuration includes 6 inlets, each with a flow rate of 0.2 ml min^−1^, the density and viscosity coefficient of gastric fluid were set to 1,000 kg m^−3^ and 60 MPa·s, respectively, and no-slip boundary conditions were applied to all channel walls [51].

### Characterization of the circuit

The theoretical operational duration of the circuit is derived from the power consumption report. In the working station, the average current is 468.4 μA, while in the sleep station, the average current is 20.2 μA. The thermal test of the circuit was conducted in operational mode at an ambient temperature of 24.5 °C. The circuit’s temperature increased by 1 °C over 30 min, which is insufficient to cause thermal damage to GI tissue interfaces. The temperature sensor was calibrated after capsule integration and tested in a constant temperature- and humidity-controlled chamber. Since the chamber’s temperature rise is continuous, the trend shown in Fig. 3D exhibits a linear increase, with all deviations falling within reasonable limits.

### Hydrogel skin formation

The uncoated capsule cap and body were initially cleaned with ethanol and dried under nitrogen flow. To enhance the hydrophilicity of the polymer, the samples were subjected to plasma treatment using a plasma cleaner (PCE-80, Hefei Crystal Material Technology Co., Ltd.) for 1.5 min. Subsequently, the treated samples were immersed in an ethanol solution with 10 wt% benzophenone for 10 to 15 min. After removing excess solution from the surface, the samples were then immersed in a pre-gel solution composed of 20 wt% hydrogel monomers (acrylamide; Aladdin, Shanghai, China) and 1 wt% ammonium persulfate (Aladdin, Shanghai, China) dissolved in deionized water. The pre-gel solution was degassed for 5 min prior to preparation. In the end, the pre-gel solution bath was exposed to ultraviolet irradiation for 60 min. Then, any unreacted reagents were meticulously eliminated through rinsing with deionized water, utilizing an orbital shaker (Microplate Shaker, VWR) for a continuous period of 24 h.

To visualize the hydrogel skin (Fig. 2A to D), both coated and uncoated samples (20 mm × 20 mm × 1 mm), prepared by molding from Ecoflex 00-30/NdFeB (50 wt%), were immersed in an aqueous fluorescein solution (Aladdin, Shanghai, China) prior to imaging.

### SiO_2_ coating of magnetic particles

The Stöber method was employed to coat NdFeB microparticles with a layer of silicon dioxide (SiO_2_) by hydrolysis and polycondensation of tetraethyl orthosilicate (TEOS, Aladdin, Shanghai, China) [52,53]. Initially, 40 g of NdFeB microparticles was dispersed in 1,000 ml of ethanol under vigorous stirring to prevent precipitation. Subsequently, 60 ml of 29% ammonium hydroxide was slowly added to the mixture, followed by 2 ml of TEOS. The mixture was stirred at room temperature for 12 h and then allowed to stand for an additional 12 h to ensure complete reaction. Upon completion of the reaction, anhydrous ethanol was added, and the mixture was centrifuged (HC-3018, Anhui USTC Zonkia Scientific Instruments Co., Ltd.) at 7,000 rpm for 5 mins. The supernatant was decanted, and this washing procedure was repeated 3 times to obtain the final NdFeB@SiO_2_ product.

### Assessment of biocompatibility

The Caco-2 cells were used as the model cell line for evaluating cell viability. For in vitro cytotoxicity testing, hydrogel-conditioned medium was prepared by incubating 20 mg of each sample in 1 ml of Minimum essential medium (MEM, catalog number 11095-080, Gibco) at 37 °C for 1, 4, and 7 days.

Caco-2 cells were cultured in MEM supplemented with 20% fetal bovine serum (10091-148, Gibco), 1× non-essential amino acids solution (11140-050, Gibco), and 1% penicillin/streptomycin (P7630, Solarbio). Cells were seeded in 96-well plates and subsequently treated with the conditioned medium derived from the samples. The cells were then treated with the sample-conditioned medium and incubated at 37 °C for 48 h in 5% CO_2_ atmosphere. Cells grown in unconditioned MEM served as the positive control, while cells exposed to 70% ethanol for 30 min after being cultured in MEM were used as the negative control. Cell viability following exposure to the different materials was assessed using the Live/Dead cell imaging kit (40747ES76, YEASEN). Fluorescence microscopy images were acquired using a high-resolution microscope. Live and dead cells were quantified using the integrated hybrid cell count analysis module in ImageJ, and the ratio of live cells to total cells was calculated to determine Caco-2 cell viability.

The CCK-8 kit (Beyotime, China) was employed to assess the viability of cells in the samples. At each time point (1, 4, and 7 days), 10 μl of CCK-8 solution was added per 100 μl of medium in each well, followed by incubation in a cell incubator for 1 h. Subsequently, absorbance values at 450 nm were measured using a microplate reader, and cell viability was calculated accordingly.

## Data Availability

All data are available in the main text or the Supplementary Materials.

## Supplementary Materials

Supplementary 1 Figs. S1 to S12 Notes S1 to S6 Tables S1 and S2 Movies S1 to S6
