# Supplementary material for: A Low-Friction Capsule Robot with Drive–Control–Sensing Integration for Gastrointestinal Lesion Detection
Source: Research (Wash D C). 2025 Aug 4;8:0807. doi: 10.34133/research.0807 (PMC12320490; doi:10.34133/research.0807)
Supplement: Supplementary 1 — Figs. S1 to S12 Notes S1 to S6 Tables S1 and S2 Movies S1 to S6 [file research.0807.f1.zip › Supplemental Information.docx]

Supplementary Materials for

**A Low-Friction Capsule Robot with Integrated Drive-Control-Sensing for Gastrointestinal Lesion Detection**

Ziying Wang *et al.*

*Corresponding author. Email: sjy2078@126.com

**This PDF file includes:**

Figs. S1 to S12

Tables S1 to S2

Supplementary Text S1 to S6

References (1 to 10)

**Other Supplementary Materials for this manuscript include the following:**

Movies S1 to S6

**Supplementary Fig.s**

**Fig. S1** The photo of magnetic actuation system.

**Fig. S2** Cyclic voltammetry (CV) cycles of polyaniline.

**Fig. S3** Experimental diagram for the quantification of the magnetic flux density of permanent magnet.

**Fig. S4** Quantification of magnetic forces and torques with different *l_lag_* when fixed *l_mag_*.

**Fig. S5** Coordinate system and via-points discretization for curved routes traversing.

**Fig. S6** Strategies for tube model traversing.

**Fig. S7** Quantification of Young’s modulus for capsule head with different magnetic particle content.

**Fig. S8** Fabrication details of the capsule robot.

**Fig. S9** Characterization of coating materials

**Fig. S10**. Cell viability tests of hydrogel skin coated material samples.

**Fig. S11** Mechanistic analysis of capsule robots overturning gastric wall folds.

**Fig. S12** Mechanistic analysis of capsule robots traversing curved small intestine.

**Supplementary Table**

**Table S1** Nomenclature of the variables.

**Table S2** Comparison with similar types of the capsule robots for monitoring the pH in GI tract.

**Supplementary Notes**

**Note 1.** Fabrication details of the capsule robot.

**Note 3.** Evaluation and improvement of biocompatibility.

**Note 4.** Multimodal motion of capsules.

**Note 5.** Force modeling and analyses.

**Note 6.** Mechanistic analysis of capsule robots overturning gastric wall folds.

**Note 7.** Mechanistic analysis of capsule robots traversing curved small intestine.

**Supplementary Movies**

**Movie S1** Velocity test of capsule robot movement in straight porcine small intestine.

**Movie S2** Multimodal motion of capsule robot in a dry stomach model.

**Movie S3** Motion efficiency in gastric model

**Movie S4** Navigational capability of capsule robot in confined luminal spaces

**Movie S5** Multimodal motion of capsule robot in *ex vivo* porcine stomach.

**Movie S6** The intestinal navigation capability of capsule robot in *ex vivo* porcine small intestine.


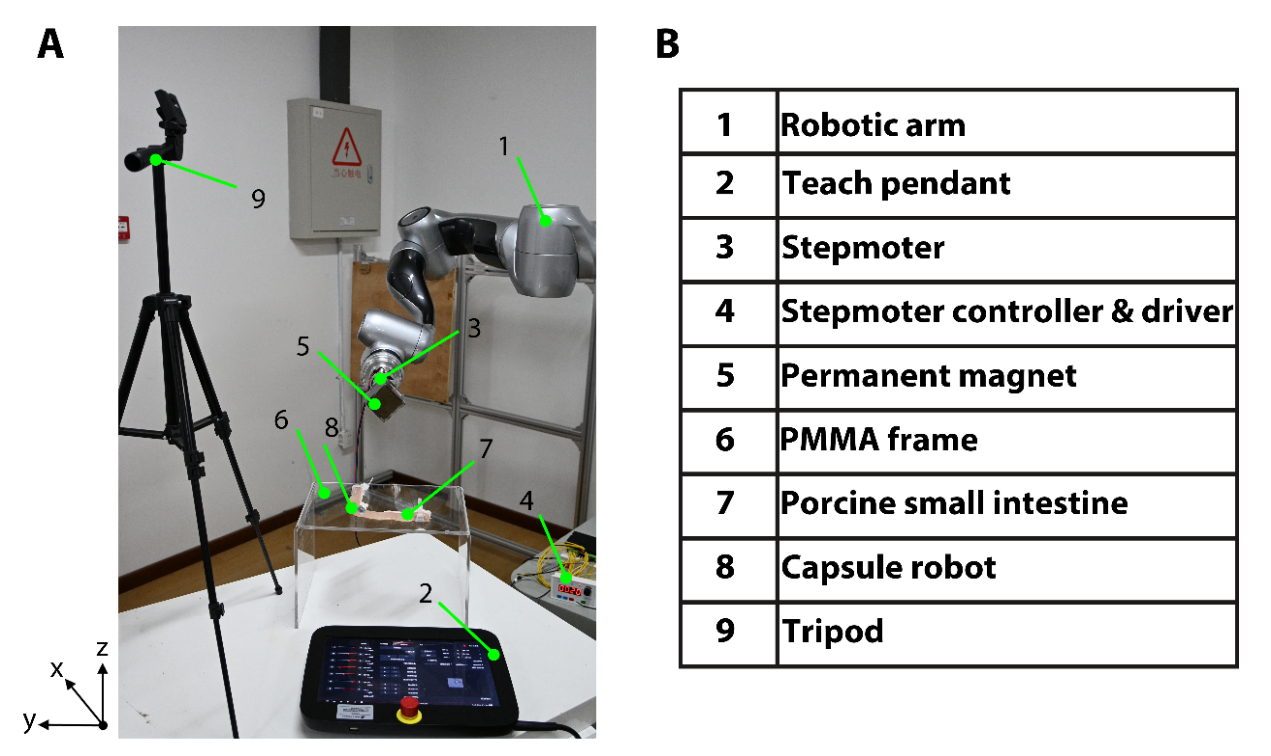


1. **The photo of magnetic actuation system.**


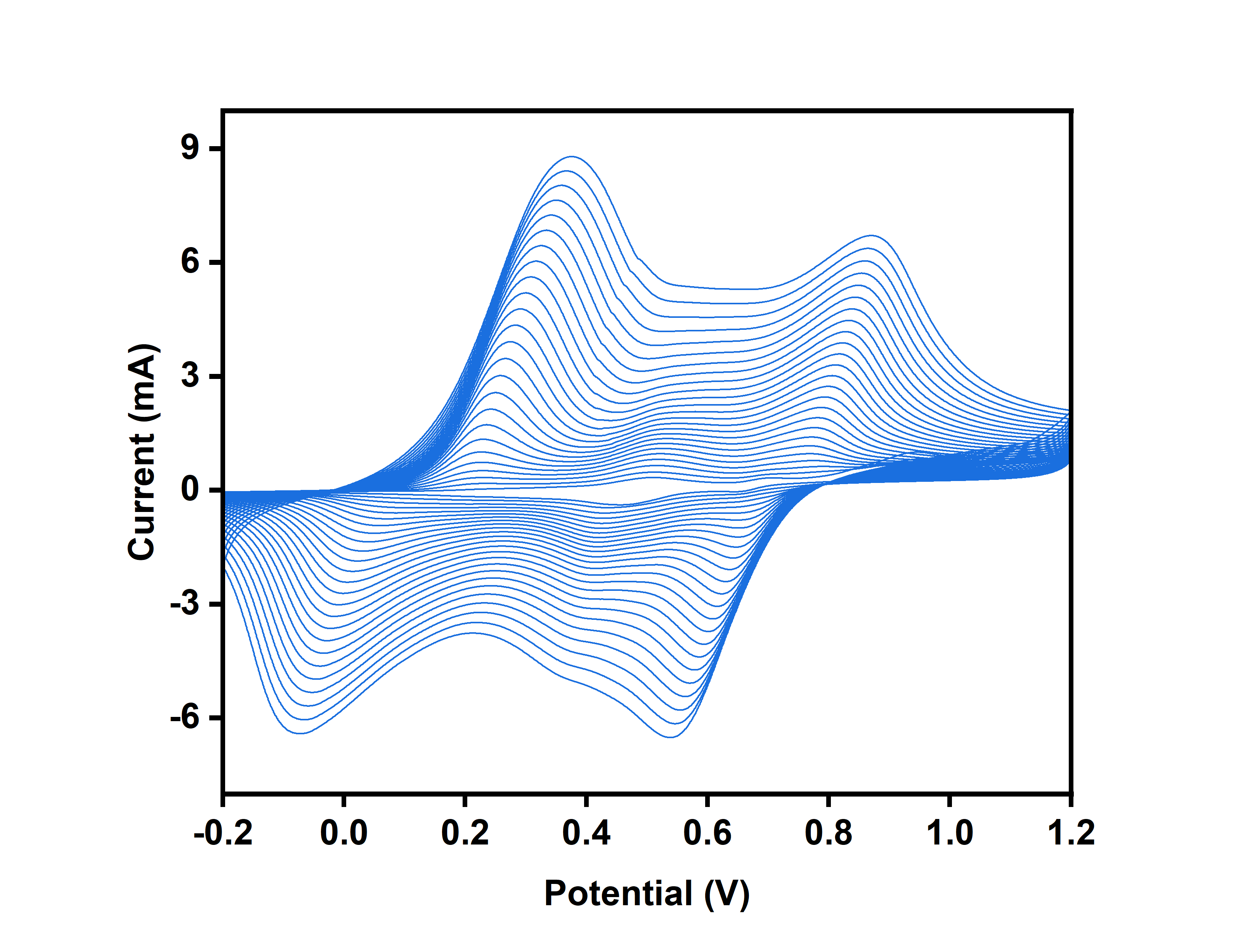


1. Cyclic voltammetry (CV) cycles in the potential range of -0.2-1.2 V for 50 segments in a 0.1 M aniline aqueous solution containing 1 M HCl.

**
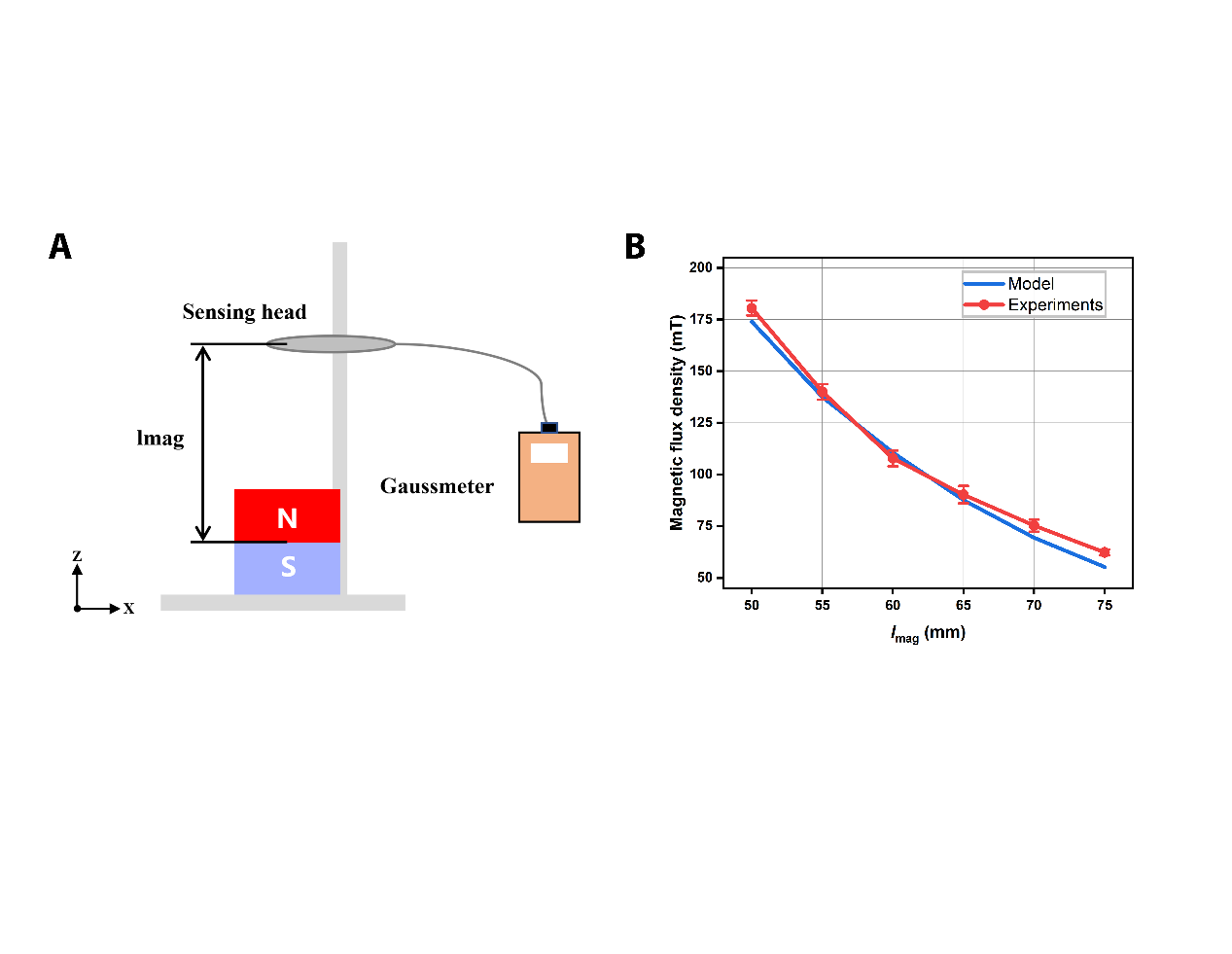
**

1. **Experimental diagram for the quantification of the magnetic flux density of permanent magnet.**

(A) Schematic of the setup to measure the magnetic flux density and magnetic forces. (B) Consistent modeled results and experimental measurements of magnetic flux density. The data are presented as mean values ± standard deviation for n = 5.


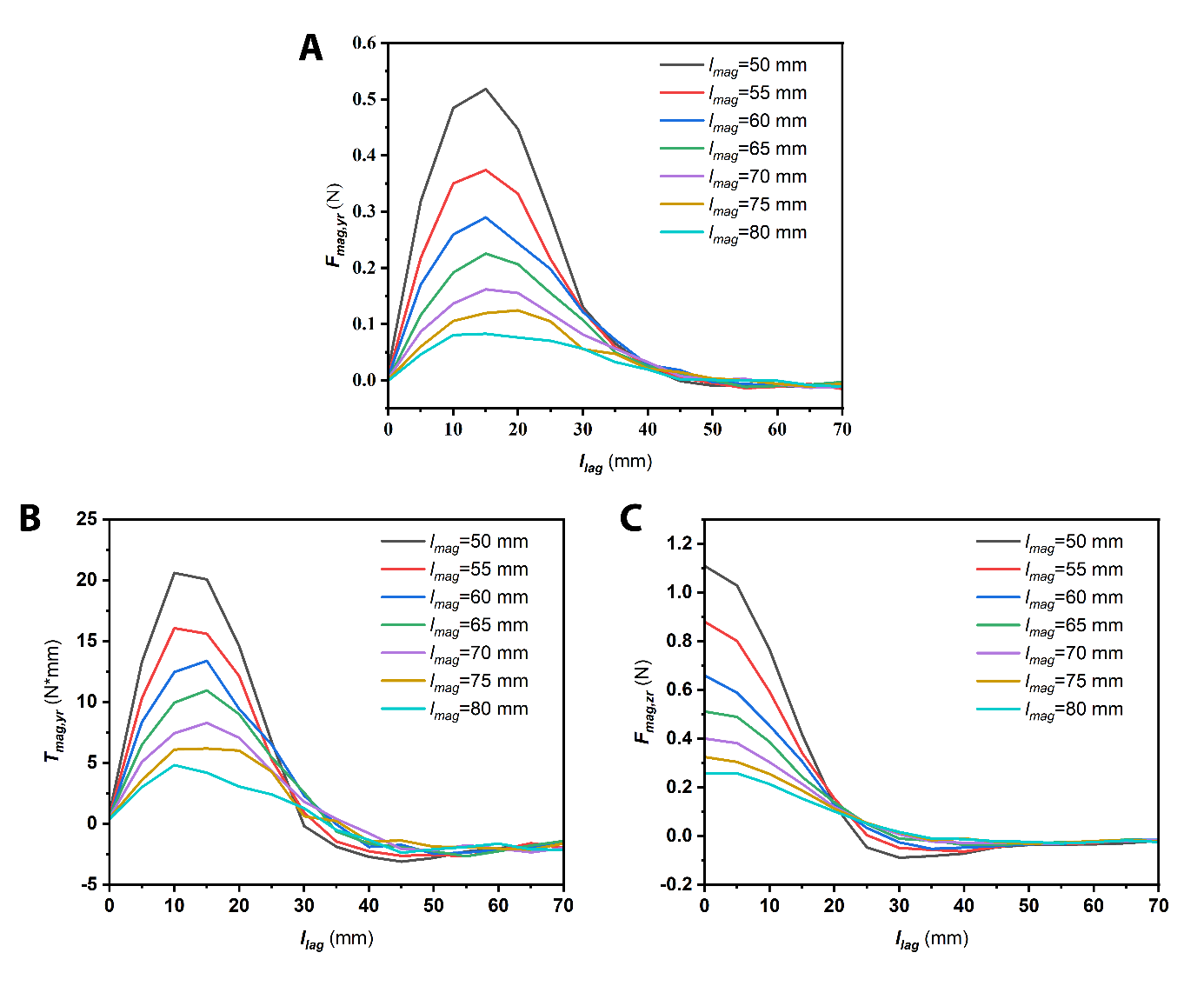


1. **Quantification of magnetic forces and torques with different *l_lag_* when fixed *l_mag_*.**

(A) Variation of the maximum magnetic force along the *y_r_*-axis *F_mag,yr_* for different distances between the robot and the magnet along with the *y_r_*-axis. Maximum *F_mag,yr_* is achieved when the magnet leads the robot by around half of the size of the robot, i.e., 15 mm.

(B) Variation of magnetic torque around the *y_r_*-axis *T_mag,yr_*. Maximum *T_mag,yr_* is achieved when the *l_lag_* = 10 mm ahead of the robot.

(C) Variation of magnetic force along the *z_r_*-axis *F_mag,zr_*. Notably that *F_mag,yr_*, *F_mag,zr_* and *T_mag,yr_* tend to 0 when *l_lag_* > 40 mm.

**
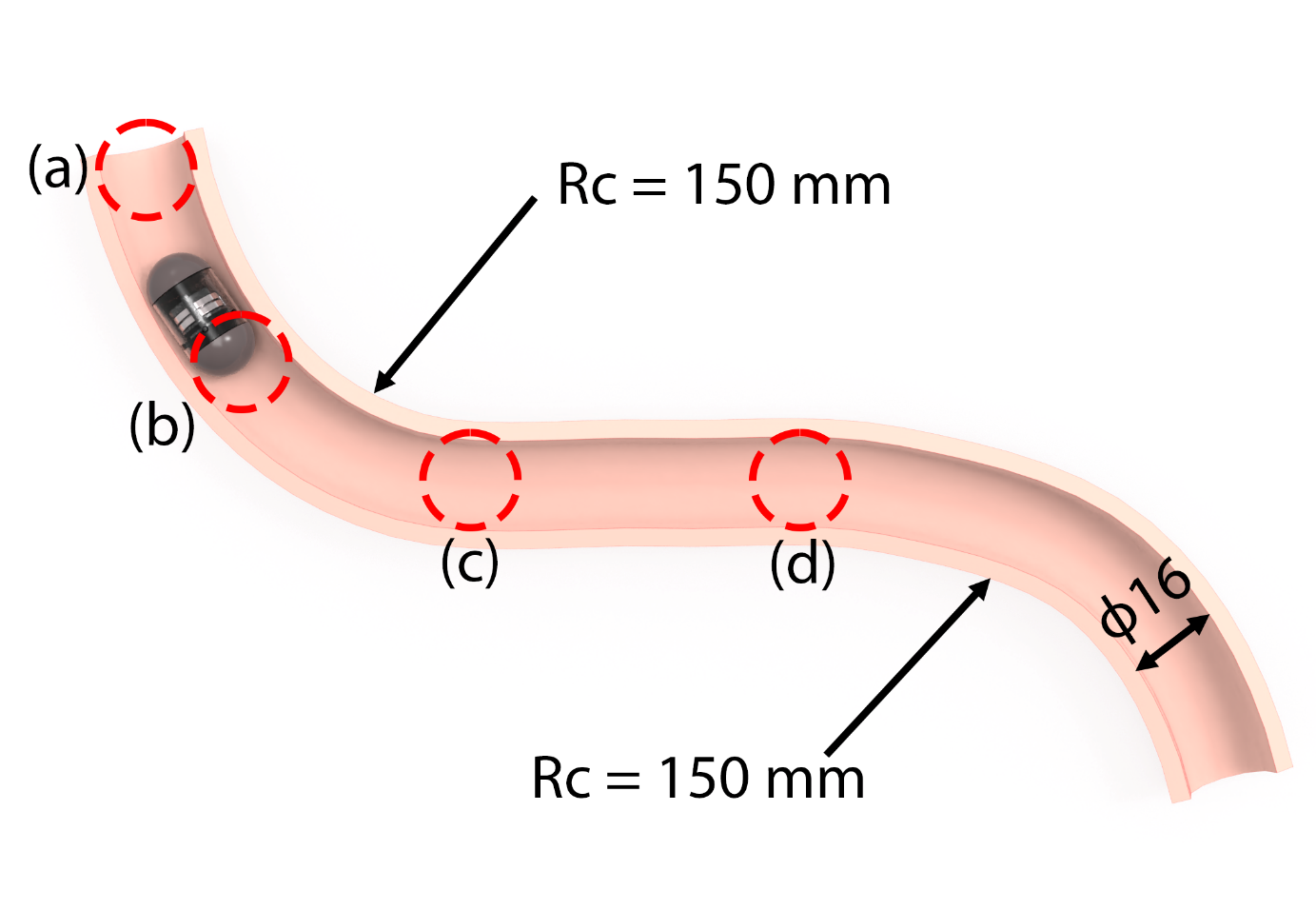
**

1. Coordinate system and via-points discretization for curved routes traversing.


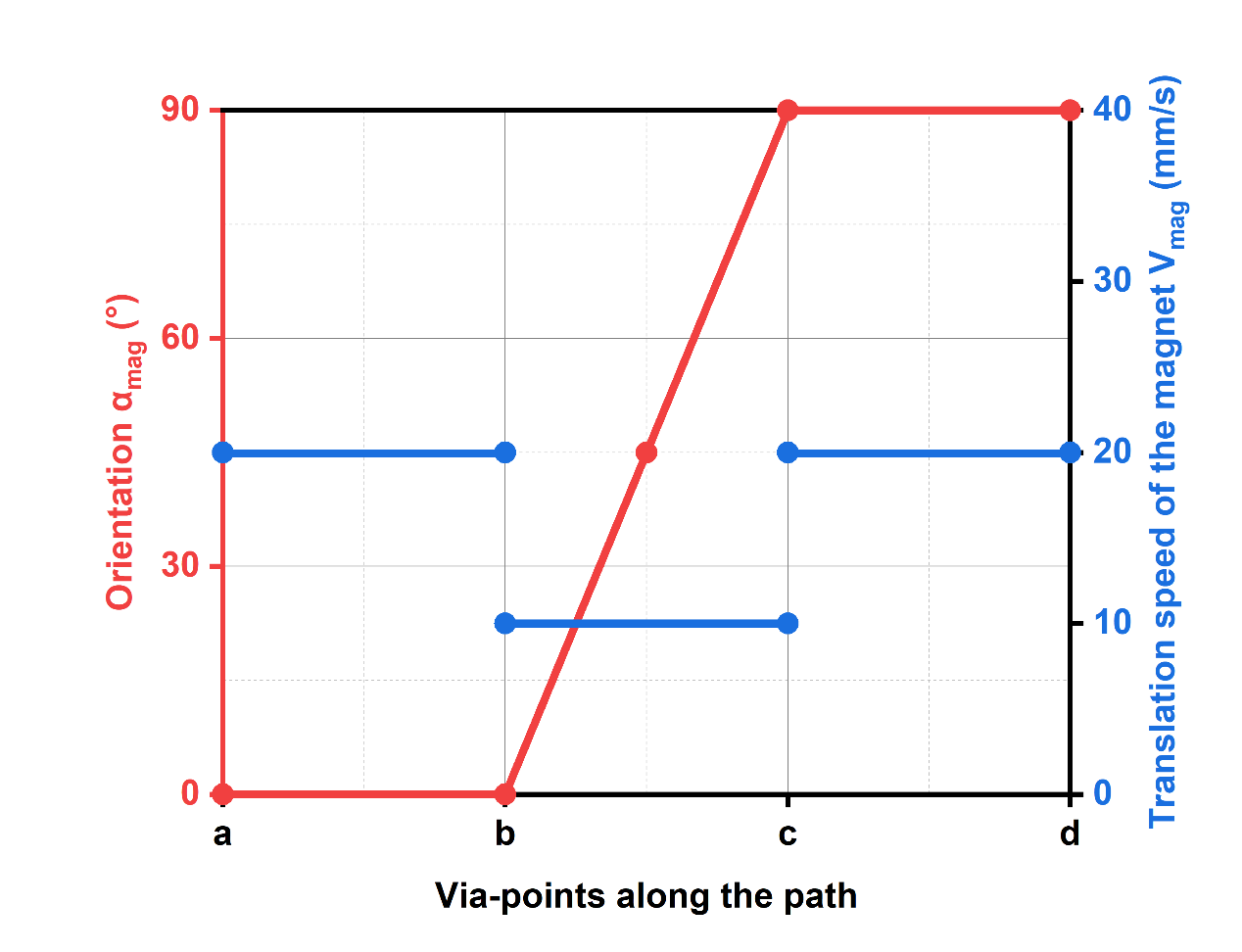


1. Strategies for curved routes traversing.

**Table S1. Nomenclature of the variables**

| **Variables** | **Explanation** |  |
| --- | --- | --- |
| $F_{mag}$ (N) | | Magnetic forces |
| $T_{mag}$ (Nm) | | Magnetic torques |
| $f_{mag}$ (rad·min^-1^) | Rotation frequency of the magnet |  |
| $v_{mag}$ (mm·s^-1^) | Translation speed of the magnet |  |
| $v_{r}$ (mm·s^-1^) | Translation speed of the robot |  |
| $l_{mag}$ (mm) | Distance between the magnet and the robot along the *z_r_*-axis |  |
| $l_{lag}$ (mm) | | Distance of robot’s lag to magnet along the *y_r_*-axis |
| $F_{react}$ | Reaction force |  |
| $T_{react}$ | Reaction torque |  |
| $E_{r}$ (MPa) | Young’s modulus of the robot material |  |
| $B\left( P_{a}^{r} \right)$ | Magnetic flux density generated by the actuation magnet with the magnetic moment of $m_{a}$, and $P_{a}^{r}$ is the vector  pointing from the actuation magnet to the robot |  |

**Table S2. Comparison with similar types of the capsule robots for monitoring the pH in GI tract.**

| **Function** | **pH** | **Sensitivity** | **Detection method** | **Average movement speed** | **References** |
| --- | --- | --- | --- | --- | --- |
| Sensing only | 1-9 | 74.37 mV pH^-1^ | Passive mode | - | [1] |
|  | 1-8 | 43.2 mV pH^-1^ | Passive mode | - | [2] |
|  | 2-8 | 44.4-59.6 mV pH^-1^ | Passive mode | - | [3] |
|  | 1-6 | - | *In situ* detection | - | [4] |
| Integrated Drive-Control-Sensing | 2-8 | 60.67 mV pH^-1^ | Multimodal motion | 12.79 mm s^-1^ | This work |

**Supplementary Note 1. Fabrication details of the capsule robot**

**1.1 Fabrication of capsule heads**

The majority of the risk of damage to the GI tract by the capsule robot is attributed to the movement of the capsule’s head during transit. As magnetic soft composites have excellent performance in terms of deformability, controllability, and flexibility,^[5-8]^ we introduce the magnetic soft composites with embedded neodymium-iron-boron (NdFeB) microparticles into the soft capsule head design. The capsule robots' movement characteristics of the magnetic soft capsule head are directly determined by the magnetization strength, which is closely associated with the mass fraction of NdFeB particles in its composites (Fig. S7A). Maintaining a low Young's modulus of the capsule head reduces capsule damage to the intestinal wall. In fact, either too high or too low content of NdFeB particles is not suitable for practical applications because the former will affect the Young's modulus of the material, which in turn will affect the final capsule fabrication results, such as poor tensile properties, difficulty in de-molding, and non-uniform distribution of the magnetic particles after mixing, whereas the latter will affect the manipulation properties of the capsule. To experimentally explore which design could fulfill such a requirement, we tested a class of capsule head prototypes with different Young's modulus $E_{r}$ from 0.05MPa to 0.25MPa (Fig. S7B). As shown in Fig. S7C, by testing the relationship between different magnetic particle contents and *l_mag_*, it was found that the magnetic particle content at 50wt% just meets the requirements for use.

**
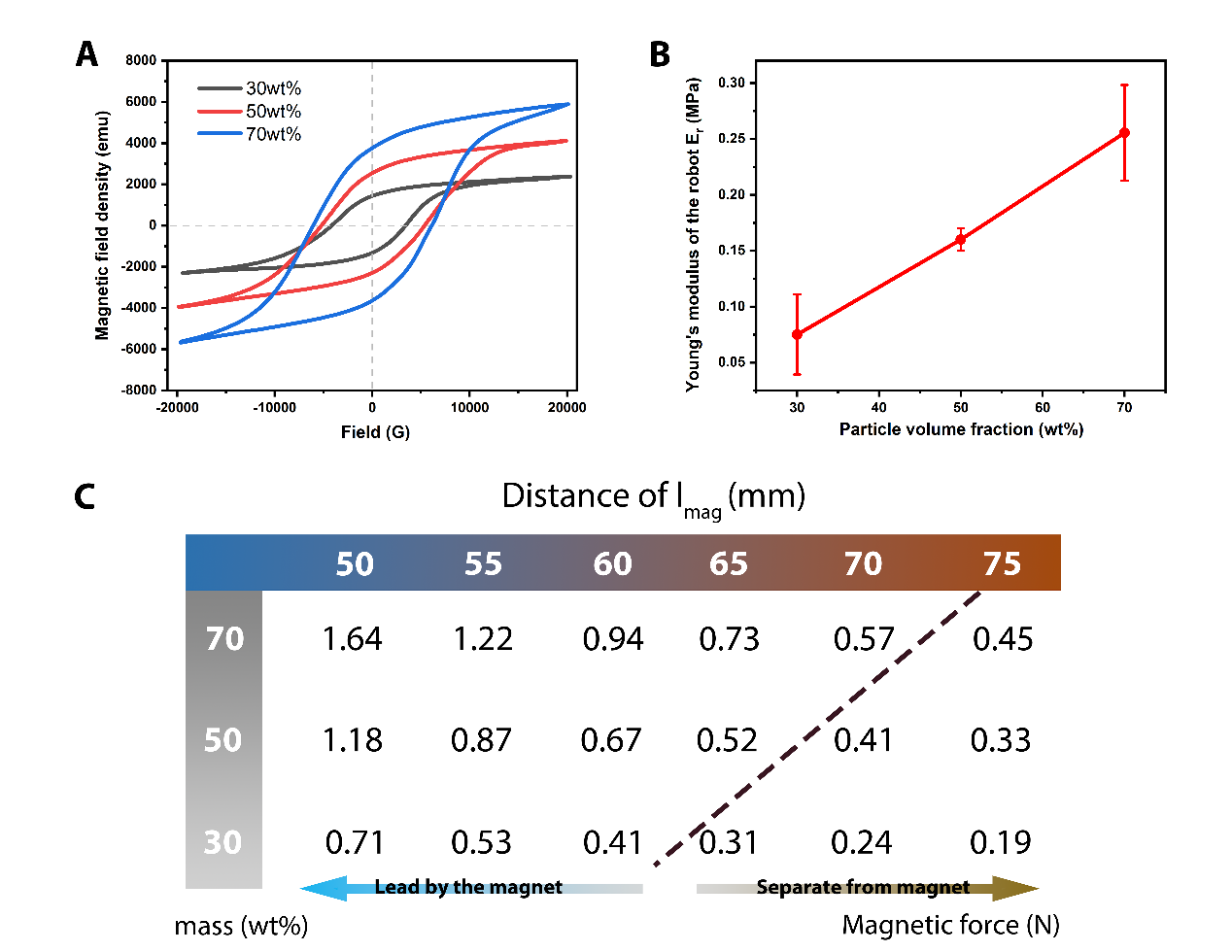
**

1. **Quantification of Young’s modulus for capsule head with different magnetic particle content.**

(A) Magnetic hysteresis loops of the magnetic soft composites containing NdFeB microparticles and Ecoflex 00-30.

(B) Characterization of *E_r_* for magnetic composite with different mass ratios of NdFeB. The ratios of the NdFeB are indicated in the Fig. The data are presented as mean values ± standard deviation for n = 3.

(C) Quantification of magnetic particle content and $l_{mag}$. Magnetic force on capsule along the $z$-axis when the distance of the permanent magnet is increased from 50mm to 75mm.

**1.2 Fabrication of the capsule robot**

There are three issues that need to be addressed during the fabrication of the capsule robots: 1) Uniformity and standardization of capsule shape, capsule size and head curvature parameters deviate greatly when utilizing commercially available capsule molds of different shapes. 2) Multichannel fluid cavity will clog during curing of hydrogel coatings. 3) Mitigating the impact of strong magnetic fields during magnetization on the movement of charges within the electrical circuits.

Firstly, we made a capsule mold using 3D-printing according to the designed capsule size, mixed Ecoflex 00-30 with NdFeB particles 1:1, degassed and filled into the capsule head mold, cured and de-molded in an oven to obtain the magnetic capsule head and capsule body (Fig. S8A-C). Next, the surface of the cured robots was treated with an ethanol solution containing benzophenone. The photo initiators within this organic solution were absorbed into the robot's surface through swelling-driven absorption. Subsequently, the treated body was immersed in a hydrogel monomer solution (Fig. S8D). Upon exposure to ultraviolet (UV) radiation, the hydrogel monomers underwent polymerization initiated by the hydrophilic initiators and were covalently grafted onto the surface-bound elastomers via the activated benzophenone. This resulted in the formation of a thin interpenetrating hydrogel layer on the surface (Fig. S8E). The magnetic capsule head was then loaded into a mold and normalized under a strong magnetic field of 1.8 T (Fig. S8F), and the sensors and circuits were encapsulated in the capsule body (Fig. S8J). Finally, the magnetic capsule head, capsule body, and microfluidic multichannel chamber were concentrically bonded together using a medical-grade adhesive (Loctite 435, Henkel Investment Co., Ltd., China) to obtain the capsule robot with hydrogel skin.


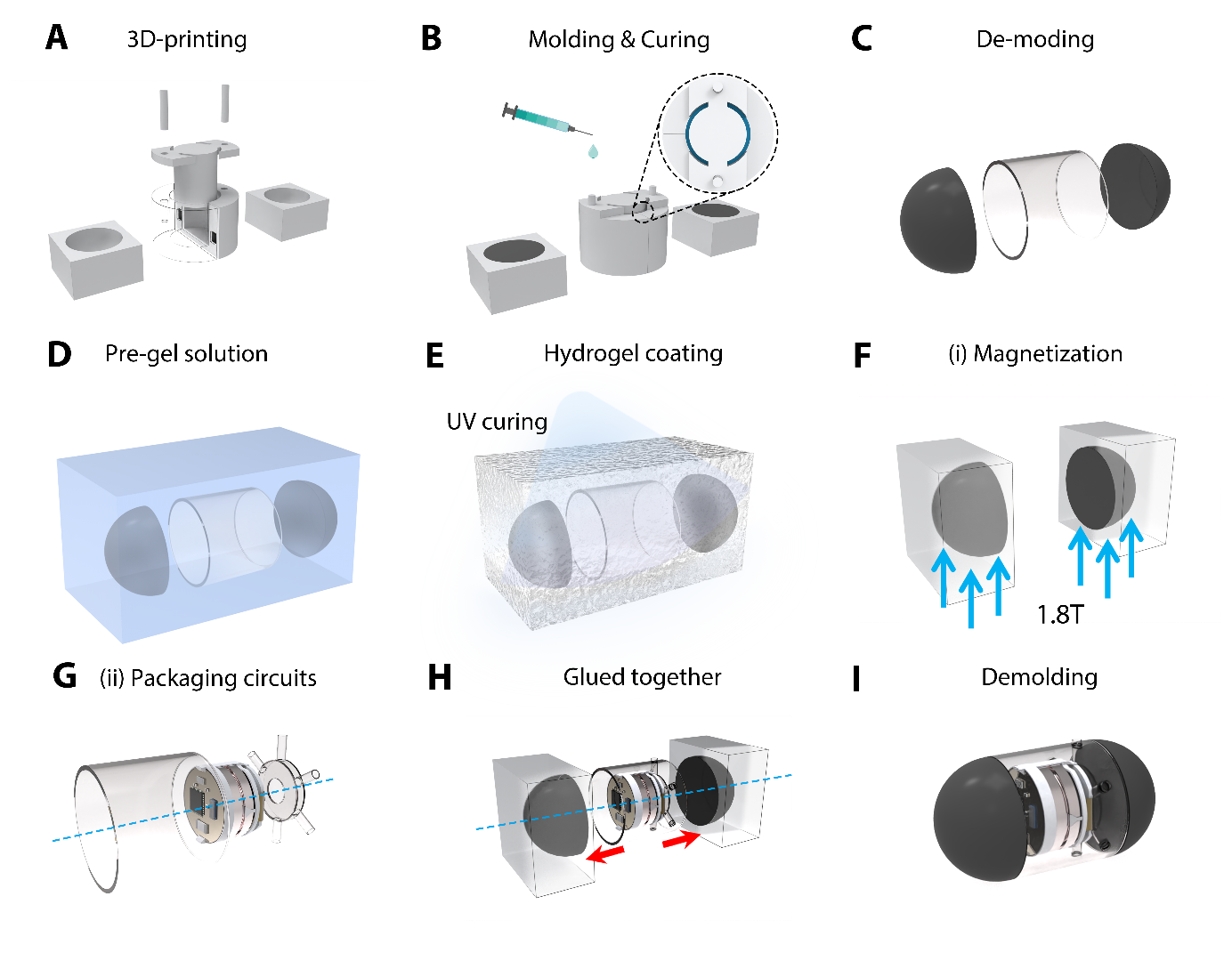


1. **Fabrication details of the capsule robot.**

(A) 3D-printing of the capsule head and body mold.

(B) Fabrication of the capsule head and body.

(C) De-molding to get the capsule head and body.

(D) Immersion in hydrogel solution.

(E) Hydrogel skin obtained by UV curing.

(F) Magnetization of the robot under the 1.8 T uniform magnetic field in 3D-printed molding.

(G) Encapsulated circuitry in capsule body.

(H) Glued capsule head and body together.

(I) De-molding and obtained the capsule robot.

**Supplementary Note 2. Evaluation and improvement of biocompatibility**

**2.1 Coatings to improve biocompatibility**

NdFeB is not biocompatible due to its corrosive nature.^[9]^ After coating NdFeB microparticles with silica to improve the biocompatibility, The magnetic hysteresis was first quantified for both bare and coated particles. The comparison revealed negligible differences in magnetic properties between the samples (Fig. S9A). To simulate gastric conditions, uncoated and coated NdFeB particles were subjected to leaching tests in 0.2 mM HCl solution (pH 3.7) for 3 days. No visible change was observed in the silica-coated particles owing to the presence of the protective silica layer, whereas the uncoated particles were highly oxidized, turning the color of the solution yellow, which demonstrates that silica coating improves the biocompatibility of magnetic capsule heads (Fig. S9B).

We then considered the effect of silica coating and hydrogel skin on the mechanical properties of the capsule head separately. Dog bone–shaped specimens with known dimensions based on Ecoflex 00-30/NdFeB with different particle concentrations were prepared by molding, and made specimens with hydrogel skin. Tensile tests were carried out using a mechanical testing machine and the results showed that the silica coating and hydrogel skin did not contribute to the overall mechanical properties of the specimens, as reported in the literature (Fig. S9 C, D).


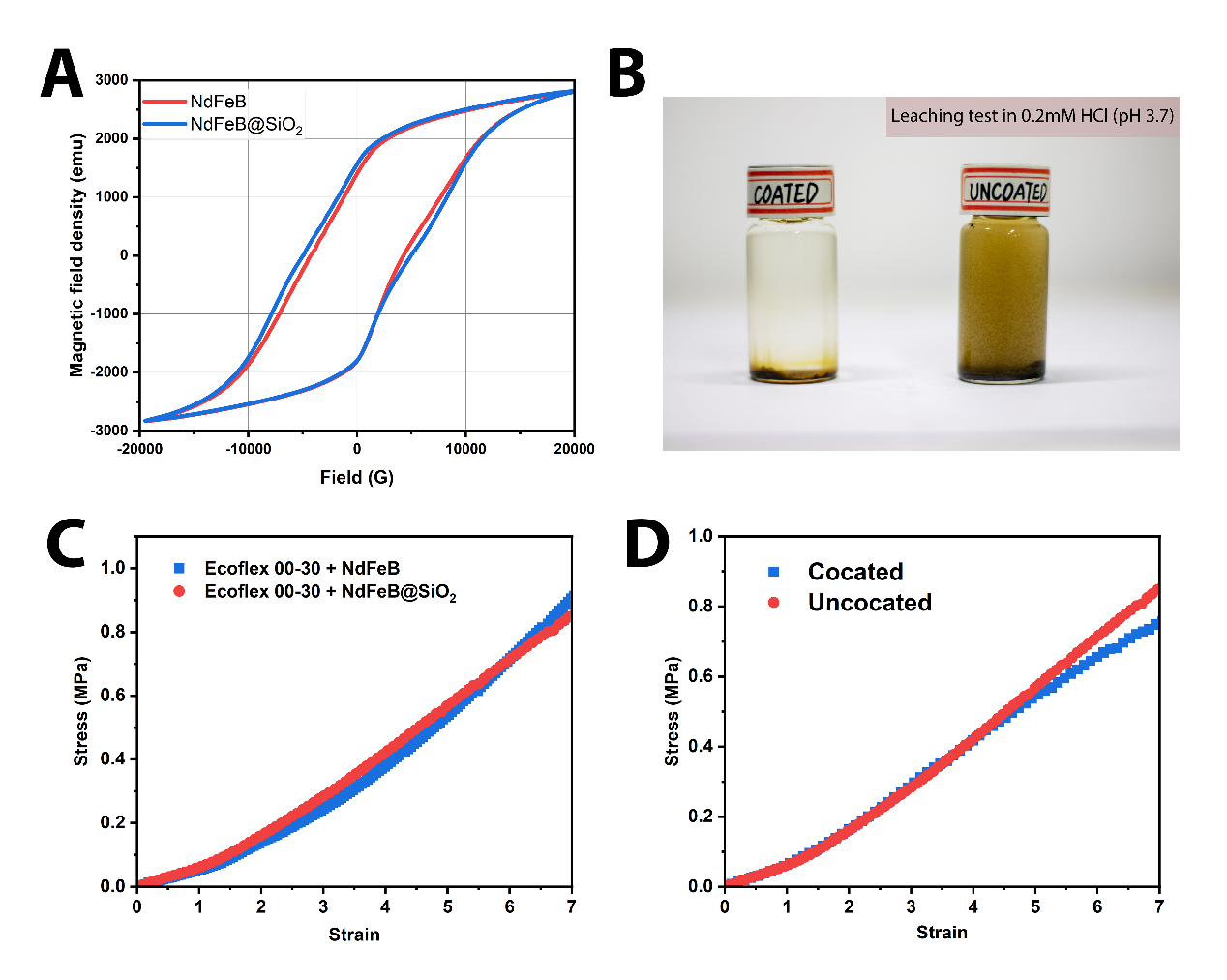


**Fig. S9. Characterization of coating materials.**

(A) Magnetic hysteresis of the bare NdFeB microparticles and the silica-coated particles. Sample weights: 40 mg.

(B) Leaching test of both uncoated and coated NdFeB particles in 0.2 mM HCl solution (pH 3.7) for 3 days. No visible change was observed in the silica-coated particles owing to the presence of the protective silica layer, whereas the uncoated particles were highly oxidized, turning the color of the solution yellow.

(C) Stress-Strain curves before and after coating silicon.

(D) Stress-Strain curves of coated and uncoated hydrogel skins.

**2.2 Biocompatibility**

The capsule head material was carefully selected to ensure safe operation. To prevent potential damage to gastric and intestinal walls during propulsion, soft materials were employed for the fabrication of the capsule's head. PDMS is known for its excellent biocompatibility and lack of acute cytotoxicity,^[10]^ was considered despite its limitations in blood-contacting applications due to rapid protein adsorption and relatively high platelet adhesion.^[11-13]^Therefore, PDMS cannot be used for gastric ulcer detection or for applications such as gastrointestinal bleeding. Ecoflex 00-30 was used as the main body material of the capsule head and created a hydrogel skin on its surface to further reduce secondary damage to the GI tract caused by friction and material stiffness when the capsule is turned (Fig. 4A-D).

As shown in Fig. S10A, after one day of culture, caco-2 cell survival was unchanged from the positive control. After one day and seven days of culture, only a few dead cells were detected, suggesting that the hydrogel skin coating was highly biocompatible. Fig. S10B demonstrates the quantitative analysis of the mixing contrast under fluorescence microscopy. The absorbance changes were similar in the experimental group compared to the positive control, indicating that cell viability was not affected. The results of 1-7 days of culture of the experimental groups were quantified by statistical analysis (Fig. S10C), and there was no significant difference in the survival of caco-2 cells over 1-7 days.


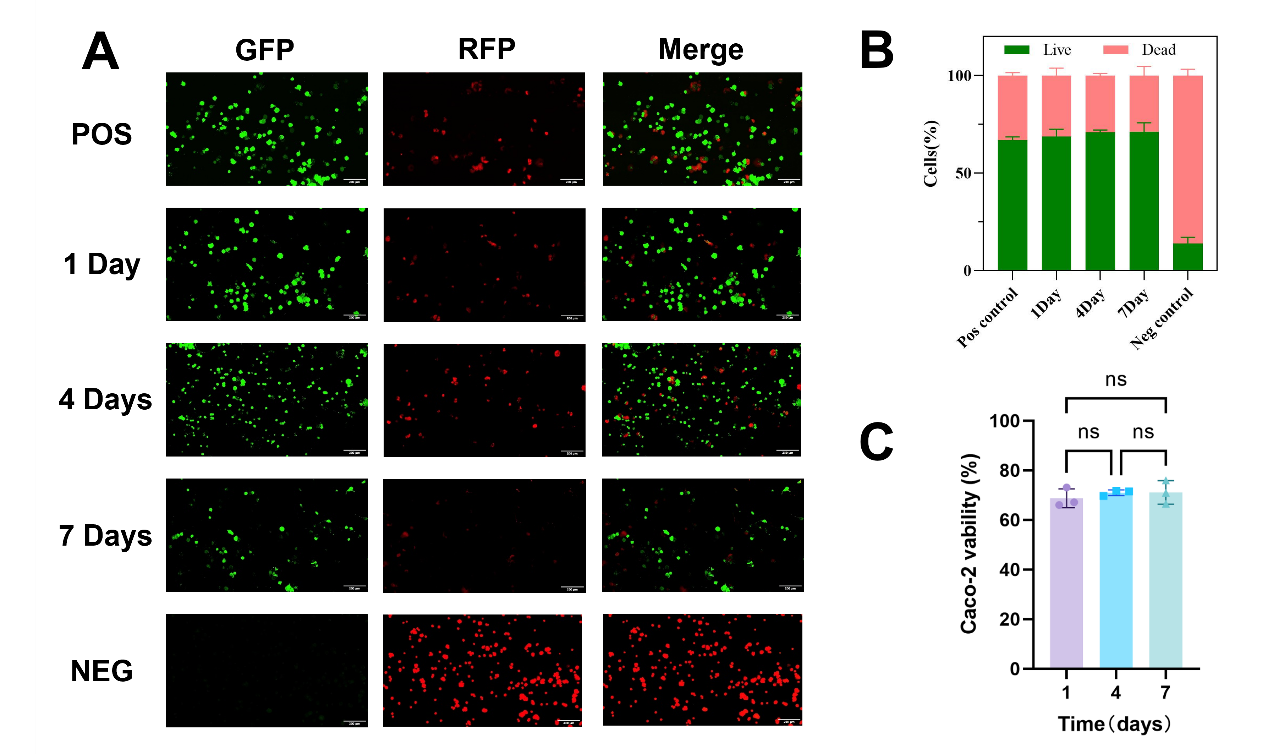


**Fig. S10. Cell viability tests of hydrogel skin coated material samples.**

(A) The live/dead staining of Caco-2 cells.

(B) Quantification of live/dead staining of Caco-2 cells.

(C) Caco-2 cell viability with varied conditioning time. The values represent the mean ± s.d. (n = 3).

**Supplementary Note 3. Force modeling and analyses**

There are three main forces affecting the movement of the capsule robot along the *y*-axis is the magnetic force *F_mag,yr_* applied to the robot by the permanent magnet along the *y*-axis, with the magnetic torque *F_mag,yr_* generated by the permanent magnet around the *y*-axis, and reaction force *F_react_* generated by the capsule coming into contact with the GI tract.

**4.1 The magnetic dipole model**

The magnetic force *F_mag_* and the torque *T_mag_* generated by the permanent magnet are obtained by modelling with the magnetic dipole model:

$F_{mag}(P_{a}^{r},m_{r})=(m_{r}\cdot\nabla)B(P_{a}^{r})$ (1)

$T_{mag}=m_{r}\times B(P_{a}^{r})$ (2)

where $m_{r}$ is the magnetic moment of the robot, and $B(P_{a}^{r})$ is the magnetic flux density generated by the actuation magnet with the magnetic moment of $m_{a}$, and $P_{a}^{r}$ is the vector pointing from the actuation magnet to the robot. All parameter quantifications are modeled by magnetic fields module in COMSOL Multiphysics 6.1.

**4.2 Quantification of the reaction force *F_react_* and reaction torque *T_react_***

Using the small intestine as the analysis scenario, where fluid resistance and intestinal drive forces are generated, we use a simplified model to describe the motion of the robot in the small intestine, i.e., ignoring the intestinal peristaltic force and fluid resistance. When the robot can perform propulsive motion, Equation (3) and Equation (4) need to be satisfied.

$F_{mag,yr}>F_{react}$ (3)

$T_{mag,yr}>T_{react,yr}=F_{react}R_{r}$ (4)

Where *F_react_* = *μN*, *N* is the normal force of the capsule robot. *R_r_* is the radius of the capsule robot, *μ* is friction coefficient of the robot's moving surface. *F_mag,yr_* here refers to the maximum achievable value, and *T_mag,yr_* refers to the one when *F_mag,yr_* is acquired.

**Supplementary Note 4. Multimodal motion of capsule robot**

As shown in Fig. 7A, the capsule robot can perform multimodal motion under the excitation of the permanent magnet, including swinging, continuous rotation and rolling by *F_mag_* to adapt different environments in GI tract. When the capsule robot entered the stomach through the cardia, the capsule swings left and right to adjust and select the direction of advance under the influence of the magnetic torques *T_mag_*, then swings to enter the crease under the traction of the gradient magnetism. When the capsule slides into the crease, it can rotate along the circumference direction by moving the permanent magnet forward, using the magnetic torque along the circumference direction to overcome the friction on the groove surface, and thus sliding along the stomach fold. When the capsule robot reaches the vicinity of the lesion area, the permanent magnet turns on the reverse rotation and slowly reduces the rotational speed, causing the capsule robot to tumble to the lesion area. When the robot reaches the lesion area, the robot locks on through a gradient magnetic field *F_mag_* and performs gastrointestinal fluid collection and analysis. When robot in the swinging and in the sliding, which response is slightly lagged, which is caused by the magnetic force of permanent *F_mag_* and magnetic torques *T_mag_* not being in the maximum position, both of two situations will not affect the robot's path selection and advancement.

**Supplementary Note 5. Mechanistic analysis of capsule robots overturning gastric wall folds**

During the movement of the capsule robot in stomach, it is not exclusively produce rolling behavior by magnetic force to roll over the stomach wall folds. In fact, there are three stages to roll over the stomach wall folds: 1) reverse rotation, 2) swinging, 3) rolling.

When the capsule robot is in the reverse rotation stage, there will be a reaction force *F_react_* on the surface of the folded projection, and the direction is tangent and vertically upward along the contact point between the capsule robot and the stomach wall, which is caused by the reverse rotation of the capsule robot (Fig. S11A). The torque produced by the magnet *T_mag_* should be greater than *T_react_*, which makes capsule robot capable of roll over the fold. However, the low coefficient of friction on the surface of the capsule robot creates a relative slip with the surface of the fold, making the *T_react_* insufficient to support the robot over the fold. Therefore, the magnet begin to guide the robot to swing so that one side of the robot can swing to the top of the crease (Fig. S11B). By the time of the swinging, *l_lag_* < 30 mm along the *y_r_*-axis to make *T_mag_*_,_*_yr_* > *T_react_*. If *l_mag_* = 50 mm, *l_lag_* should be within (25,30] mm, making *T_mag_* close to *T_react_*. Finally, gradually increase *l_lag_* to increase *F_mag_*, so that the capsule can be roll over the fold by *F_mag_*, at which point *l_lag_* should be less than 25 mm, making *T_mag_* > *T_react_* (Fig. S11C).


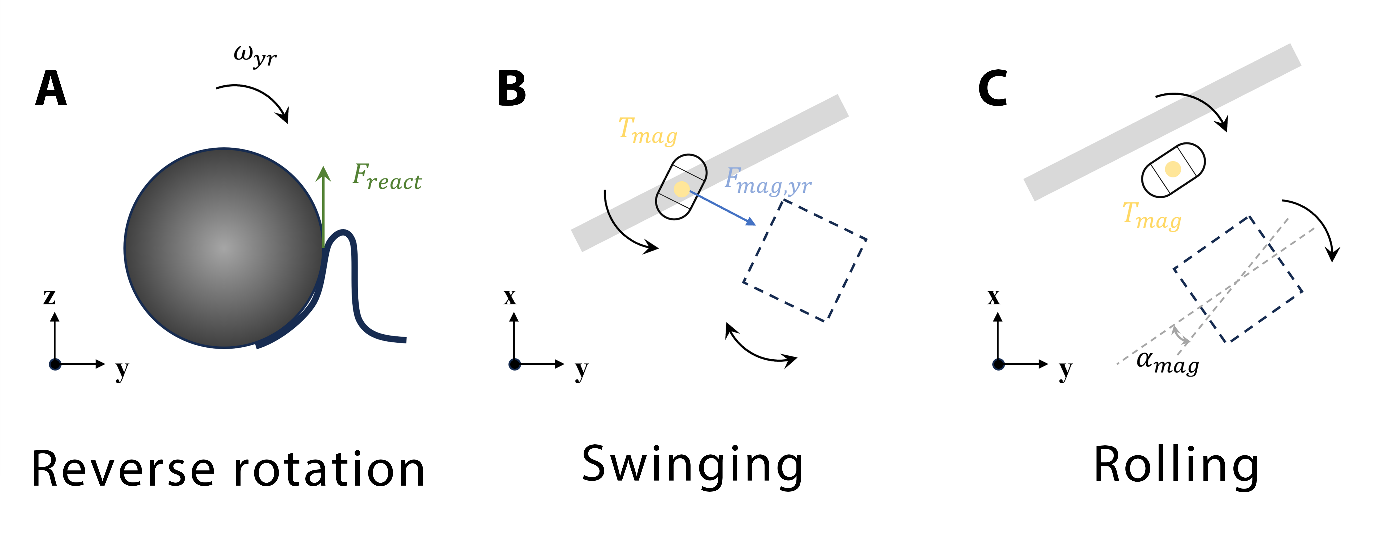


**Fig. S11 Mechanistic analysis of capsule robots overturning gastric wall folds**

(A) Rotating magnet -induced reaction force from the fold.

(B) Swinging combined with *F_react_* causes a section of the capsule to roll over the fold.

(C) Reorientation of the magnet and rolling to cross over the fold.

**Supplementary Note 6 Mechanistic analysis of capsule robots traversing curved small intestine**

When the capsule robot traverse in the curved small intestine, the capsule robot's degree of freedom is limited due to the narrow intestinal environment, so the capsule robot's movement in the intestine is only forward and backward, and the main movement mode is the rotary motion guided by the magnet.

The torques enabling the traversing of curved routes are composed of magnetic components, i.e., a) magnetic pulling force-based and b) direct magnetic torque-based, and c) the reaction-based from the lumen walls. Here we explain the modeling for each component.

**6.1 Torque from the magnetic pulling force *F_mag_***

There is always a pulling force *F_mag_* on the robot pointing to the magnet’s center when it leads the robot (Fig. S12A). *F_mag_* induces a torque *T_mag,force_* leading the robot get into (c)-(d). The maximum of *F_mag_* gives *F_mag_* = 1.2 N with *l_mag_* = 50 mm and *l_lag_* = 0 mm. The resultant magnetic torque can be approximated as

$T_{mag,force}=F_{mag}\frac{l_{r}}{2}\sin\left( \frac{\pi}{4} \right)=1.2728\times{10}^{-2}Nm$ (5)

When the magnet is not leading the robot, *F_mag_* = 0. Thus, the range of *T_mag,force_* is [0, 1.2728 × 10^-2^]. This component is constant for curved routes with different radii of curvature *R_c_*.

**6.2 Magnetic torque *T_mag_***

Due to the reorientation of the magnet, there is a rotating vector of magnetic flux density along the *x_a_*-*z_a_* plane. This vector induces the magnetic torque *T_mag_*, aligning the robot to the magnet (Fig. S12B). When the |*l_lag_*| = 10 mm, the maximum flux density along the *x_a_*-axis is *B_x,max_* = 60 mT. *T_mag,max_* can then be computed as

$T_{mag,max}=m_{r}\times B\left( P_{a}^{r} \right)=\left| m_{r} \right|\left| B_{X,max} \right|\sin\frac{\pi}{2}=6.826\times{10}^{-3}Nm$ (6)

Where *m_r_* is the magnetic moment of the robot. When the |*l_lag_*| = 40 mm, the minimum flux density along the *x_a_*-axis is *B_x,min_* = 49 mT. *T_mag,min_* can then be computed as

$T_{mag,min}=m_{r}\times B\left( P_{a}^{r} \right)=\left| m_{r} \right|\left| B_{X,min} \right|\sin\frac{\pi}{2}=5.574\times{10}^{-3}Nm$ (7)

Thus, the range of *T_mag_* is [5.574 ×10^-3^, 6.826 ×10^-3^]. This component is constant for curved routes with different radii of curvature *R_c_*.

**6.3 Torque from the reaction force *F_react_***

When the robot starts to enter the curved route, the reaction force *F_react_* from the lumen wall is applied to the robot. As shown in Fig. S12C, as the robot rotates around the positive *y_r_*-axis and tends to roll to the positive *x_r_*-axis, *F_react_* points to the negative *x_r_* -axis. The resultant torque *T_react_* leads the robot towards (c)-(d).

The mass of the capsule robot is 5.818g, and the coefficient of friction *μ* is a dimensionless number. Therefore, *F_react_* and *T_react_* can express as

$F_{react}=0.3\times5.818g\times9.18m s^{-1}=17.13 mN$ (8)

$T_{react,yr}=F_{react}R_{r}=17.13 mN\times7mm=1.1991\times{10}^{-4} Nm$ (9)

When *l_lag_* is in the effective manipulation interval, the sum of these torques are superior to the *F_react_*, and together with *T_react_*, enabling the successful curved routes traversing.

**
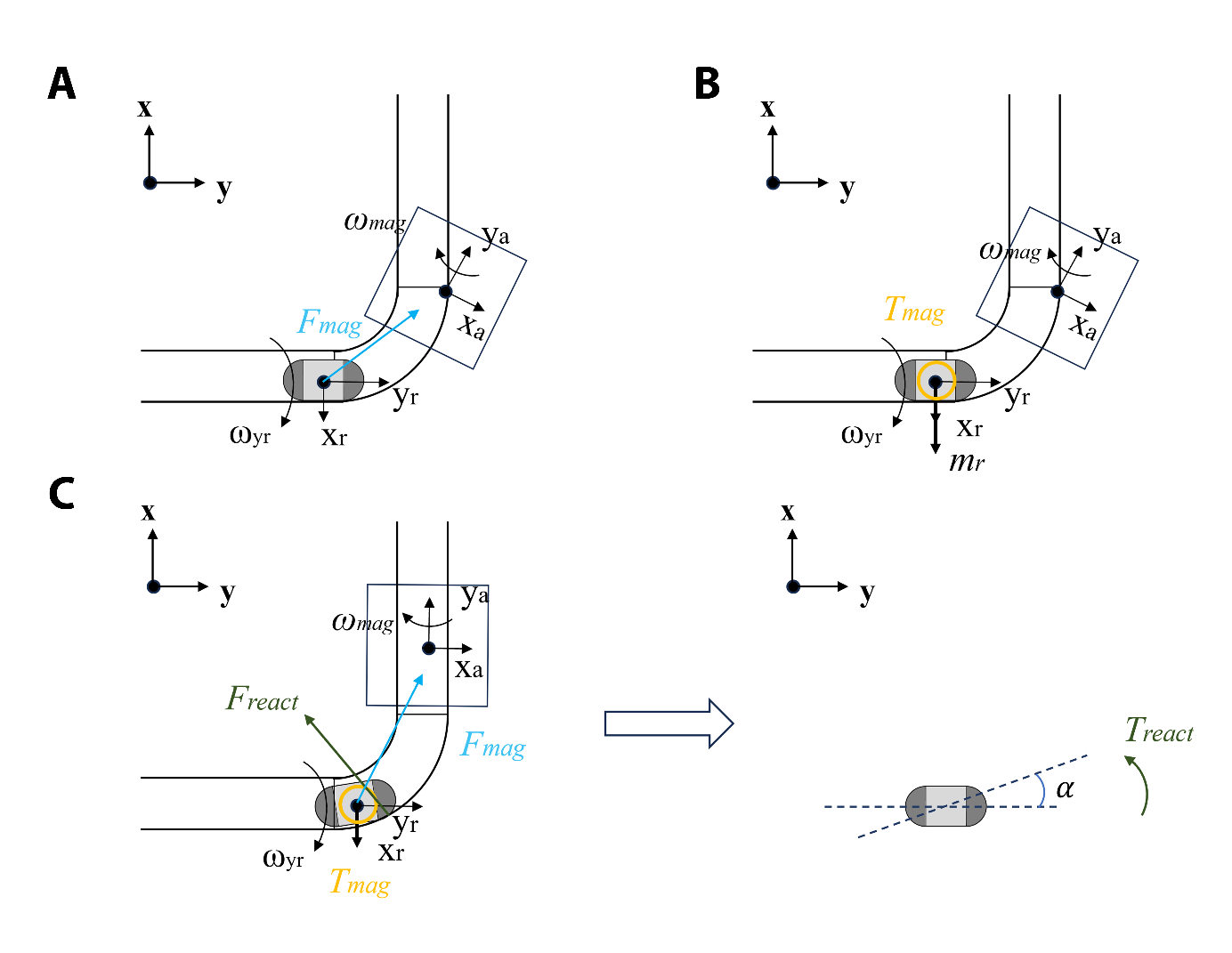
**

**Fig. S12 Mechanistic analysis of capsule robots traversing curved small intestine.**

(A) *F_mag_* induced torque leading robot get into curved segment of the small intestine.

(B) Magnetic torque due to the reorientation of the rotating magnet.

(C) Reaction force-induced torque from the intestine wall.

**References**

[1] C. Cheng, Y. Wu, X. Li, Z. An, Y. Lu, F. Zhang, B. Su, Q. Liu, *Sensors and Actuators B: Chemical* **2021**, 349, 130781.

[2] S. Gopalakrishnan, R. Thomas, S. Sedaghat, A. Krishnakumar, S. Khan, T. Meyer, H. Ajieren, S. Nejati, J. Wang, M. S. Verma, P. Irazoqui, R. Rahimi, *Biosensors and Bioelectronics: X* **2023**, 14, 100380.

[3] C. Asci, A. Sharma, R. Del-Rio-Ruiz, S. Sonkusale, *Microchimica Acta* **2023**, 190, 385.

[4] B. Hou, L. Yi, D. Hu, Z. Luo, D. Gao, C. Li, B. Xing, J.-W. Wang, C. N. Lee, R. Zhang, Z. Sheng, B. Zhou, X. Liu, *Nature Biomedical Engineering* **2023**, 7, 1242.

[5] X. Yang, W. Shang, H. Lu, Y. Liu, L. Yang, R. Tan, X. Wu, Y. Shen, *Science Robotics* **2020**, 5, eabc8191.

[6] Y. Dong, L. Wang, N. Xia, Z. Yang, C. Zhang, C. Pan, D. Jin, J. Zhang, C. Majidi, L. Zhang, *Science Advances* **2022**, 8, eabn8932.

[7] Y. Kim, H. Yuk, R. Zhao, S. A. Chester, X. Zhao, *Nature* **2018**, 558, 274.

[8] Y. Kim, X. Zhao, *Chemical Reviews* **2022**, 122, 5317.

[9] V. E. Donohue, F. McDonald, R. Evans, *Journal of Applied Biomaterials* **1995**, 6, 69.

[10] K. P. Rajan, A. Al-Ghamdi, **2014**, 111.

[11] P. Xue, Q. Li, Y. Li, L. Sun, L. Zhang, Z. Xu, Y. Kang, *ACS Applied Materials & Interfaces* **2017**, 9, 33632.

[12] K. M. Kovach, J. R. Capadona, A. S. Gupta, J. A. Potkay, *Journal of Biomedical Materials Research Part A* **2014**, 102, 4195.

[13] S. Kim, S.-h. Ye, A. Adamo, R. A. Orizondo, J. Jo, S. K. Cho, W. R. Wagner, *Journal of Materials Chemistry B* **2020**, 8, 8305.
